# Acute myocardial infarction induces sex-specific, time-dependent remodeling of the gut microbiome and intestinal immune compartment in retired breeder C57BL/6N mice

**DOI:** 10.3389/frmbi.2026.1818652

**Published:** 2026-06-08

**Authors:** Eszter Pal, Neda Omidi Arjenaki, Yao Lu, Jianguo Xia, Lorraine Chalifour

**Affiliations:** 1 Lady Davis Institute for Medical Research, Montréal, QC, Canada; 2 Division of Clinical and Translational Research, Faculty of Medicine and Health Sciences, McGill University, Montréal, QC, Canada; 3 Department of Parasitology, Faculty of Agricultural and Environmental Science, McGill University, Sainte-Anne-de-Bellevue, QC, Canada

**Keywords:** C57BL/6N, retired breeder mice, flow cytometry, gut microbiome, intestinal immunity, metabolomics, myocardial infarction, sex differences

## Abstract

**Introduction:**

The gut microbiome influences cardiovascular health through metabolite production and immune modulation. Gut microbial dynamics and cardiovascular outcomes are also shaped by biological sex. However, sex-specific responses to myocardial infarction (MI) that involve the gut microbiome and intestinal milieu remain poorly defined, particularly in older hosts. Here, we characterize gut microbiome structure and function alongside physiological and immune responses to MI across multiple tissues in aging male and female mice.

**Methods:**

MI was induced by permanent LAD ligation and confirmed by echocardiography in C57BL/6N retired breeder mice. Sham surgery (SH) and no surgery (NoSx) groups served as controls. Gut microbiota and the cecal metabolome were characterized using 16S rRNA sequencing and untargeted UPLC-MS, respectively. Immune cells in the small intestine, heart, bone marrow, and spleen were quantified by flow cytometry, and small intestinal morphology was assessed on H&E-stained sections.

**Results:**

Sex-specific differences were evident at baseline. Following MI, pronounced time- and sex-specific differences in gut microbial and immune cell populations were observed, peaking on day 3 (D3) and absent in SH and NoSx controls. Early increases in *Bacteroidaceae*, *Tannerellaceae*, and *Marinifilaceae* were present in both sexes, with sex-specific enrichment of *Bacteroidaceae* in males and *Akkermansiaceae* in females. Metabolomic analyses identified increased secondary bile acid derivatives, including cholylvaline in males and 12-oxo-lithocholic acid in females. Integration of microbiota–metabolome data revealed MI-responsive and homeostatic taxa with opposing metabolite signatures, while functional analyses indicated enrichment of propanoate and amino acid metabolism pathways. These changes were temporally aligned with acute MI-induced expansion of intestinal MHCII^+^CD11c^+^ dendritic cells and TCRαβ^+^CD4^+^, TCRαβ^+^CD8αβ^+^, and CD25^+^FoxP3^+^ regulatory T cells on D3 in both sexes. Males alone exhibited marked increases in intestinal TCRγδ^+^ T cells, while females showed increased accumulation of innate immune cells.

**Discussion:**

Convergence of peak physiological, immunological, and microbial responses on day 3 after MI reveals coordinated responses across the gut-heart axis that are fundamentally influenced by sex. Our findings highlight the need for personalized, sex-specific perioperative strategies and identify the gut microbiome as a potential therapeutic target to improve outcomes after MI.

## 1 Introduction

The intestinal tract harbors a dynamic ecosystem of trillions of microorganisms (microbiota) and their metabolites (metabolome), collectively referred to as the gut microbiome. Together, they support host intestinal health by regulating nutrient metabolism, producing bioactive metabolites, training and modulating the mucosal immune system, preserving the intestinal barrier, and limiting pathogen invasion and colonization (Sanders et al., 2021). While core microbiota remain relatively stable in healthy individuals, the function and composition of the gut microbiome are sensitive to extrinsic factors including the diet, lifestyle, sex, and age (Carding et al., 2015; Rinninella et al., 2019; Sanders et al., 2021). When the natural composition of the gut microbiome is disrupted, traditionally referred to as “dysbiosis,” its protective homeostatic function is compromised.

There is growing evidence that the gut microbiome communicates with peripheral organ systems and modulates their physiological processes, such as immune responses (Tang et al., 2017; Carrillo-Salinas et al., 2020). Notably, recent studies describe a bidirectional communication between the gut and the heart (“gut-heart axis”), mediated by microbial metabolites and immune signaling, through which the gut microbiome can influence cardiovascular health and disease progression (Yang et al., 2015; Lam et al., 2016; Brown and Hazen, 2018; Carrillo-Salinas et al., 2020; Gutiérrez-Calabrés et al., 2020; Nesci et al., 2023; Zhao et al., 2023a). Gut microbiota ablation reduces inflammatory cell accumulation in a newly injured heart, suggesting a direct link between gut microbiota and the cardiac inflammatory response (Lam et al., 2016; Zhou et al., 2018; Tang et al., 2019; Zhao et al., 2023b). Additional evidence for this two-way relationship comes from a cardiac pressure-overload model in which cardiac stress drives gut dysbiosis in a T cell-dependent manner (Carrillo-Salinas et al., 2020) and a study showing that ablation of regulatory T cells (Tregs) abolishes the cardioprotective effect of *Bifidobacterium animalis* after cardiac ischemia reperfusion (Danilo et al., 2017). Overall, these findings suggest that cardiac injury, such as myocardial infarction, may disrupt gut microbial dynamics, while gut dysbiosis and associated inflammation may impair recovery.

Myocardial infarction (MI) is a leading cause of morbidity and mortality worldwide (Krittanawong et al., 2023). MI results from obstruction of coronary blood flow that results in ischemic injury and necrosis of cardiac tissue, triggering an intense, tightly regulated inflammatory response (Fang et al., 2015; Krittanawong et al., 2023). While there is a growing body of literature examining the gut microbiome in the context of cardiovascular disease/injury, comprehensive studies incorporating the timing and extent of intestinal remodeling and a global characterization of immune populations in the gut, heart, and peripheral tissues are lacking. Moreover, age and sex as biological variables are often overlooked in many preclinical studies, despite evidence that cardiovascular disease outcomes, gut microbiome dynamics, and immune responses are age-specific and sexually dimorphic (Baars et al., 2018; Li and Kararigas, 2021; Maffei et al., 2021; Rosser et al., 2022). In fact, most studies rely exclusively on young male animals, which may not reflect age- and sex-related physiological variation observed in human populations. This is especially relevant, as older adults (≥65 years) are disproportionately affected by cardiovascular disease, make up the highest-risk group for MI, and have overall poorer prognoses (Lindsey et al., 2021; Lucà et al., 2024; Pedretti et al., 2025).

In this study, we hypothesized that MI would elicit time- and sex-dependent remodeling of the gut microbiome, accompanied by alterations in intestinal morphology and peripheral and intestinal immune cell compartments. We extend existing data by using retired breeder male and female mice to better reflect older adults who are more likely to experience an MI or ischemic event. Moreover, by directly comparing males and females, we explore how sex influences multi-organ responses to MI and highlight differences in recovery. Together, our findings demonstrate that MI triggers coordinated, sex-specific remodeling of the gut microbiome and immune cell landscapes across the intestine, heart, and hematopoietic tissues in the 7 days after injury in retired breeder C57BL/6N mice.

## 2 Methods and materials

### 2.1 Animal manipulation

All animal experiments were reviewed and approved by the Lady Davis Institute Animal Care Committee according to the guidelines of the Canadian Council on Animal Care. Retired breeder C57BL/6N male and female mice were sourced from Charles River Laboratories (Saint-Constant, QC). All retired breeders used in the study were between 10–12 months of age. Male mice were single-caged and female mice were caged in groups of 4 during a 4-week acclimation period. All mice were housed in vented polycarbonate cages (Allentown, Allentown, New Jersey) with 1/4” corncob bedding (Teklad, Madison, Wisconsin) and had a 12-hour dark/light schedule with an average temperature of 24 °C and 50% humidity. Mice were supplied ad libitum with acidified tap water and a commercially prepared, plant-based, and irradiated house diet (2018 Teklad Irradiated Global 18% Protein Rodent Diet, Madison, Wisconsin) designed to fully satisfy the nutritional needs of mice. Mice were supplied with a hut (non-recycled 100% wood pulp, BioServ, Flemington, NJ) and a nestlet (2” X 2”, pulped cotton fiber, Ancare, New York, NY). One week before surgery, mice were separated into single cages with double-distilled water delivered in glass bottles and randomly assigned to an experimental group.

A no surgery (NoSx) group was used to determine baseline values (males, n=9 and females, n=7). Two surgical groups were included to distinguish responses attributed to MI versus surgical stress alone. One group underwent sham surgery, and were euthanized after 3 days (males, n=8 and females, n=8) or 7 days (males, n=8 and females, n=8) after surgery. Another group underwent surgery to induce an MI, and were euthanized after 3 days (males, n=11 and females, n=9) or 7 days (males, n=15 and females, n=15) after surgery.

### 2.2 Surgical treatment

On the day of surgery, the surgeon randomly allocated mice not in the NoSx group to either MI-inducing or sham surgery. MI and sham surgeries were performed on the same surgical day to reduce the impact of batch effects. MI surgery involved permanent ligation of the left anterior descending coronary artery (Thibault et al., 2007). This surgery is optimal for investigating cardiac wound repair and remodeling, and models systemwide, multi-organ inflammation on the healing, post-surgery subject (Lindsey et al., 2018). MI and sham surgeries were performed as previously described (Pal et al., 2025). Briefly, mice were anesthetized with 3% isoflurane and 1.5L/min oxygen, intubated, ophthalmic ointment applied, torso fur removed, and analgesia supplied by 1mg/kg subcutaneous injection of slow-release buprenorphine. For MI surgery, the chest was opened, the heart visualized, and the coronary artery ligated. In sham surgery, the same protocol was followed but a coronary artery was not ligated. Upon closure, one drop of prepared topical analgesia (1:1 mixture of lidocaine HCl (20mg/ml) and bupivacaine HCl (5mg/ml)) was applied. Time from intubation to closure was ~20 minutes. D0 refers to events on the day of surgery. Mice were euthanized after 3 days (D3) or 7 days (D7) of observation, by anesthesia followed by cervical dislocation.

### 2.3 Echocardiography

Before euthanasia, mice from all cohorts were anesthetized with 3% isoflurane and 1.5L/min oxygen, and echocardiography was performed using a VEVO 3100 sonograph and MX550S transducer. Long axis views were analyzed using Vevo Lab proprietary software (FUJIFILM VisualSonics, Japan) for the left ventricle (LV) area and volume in systole and diastole, with pulsed-wave Doppler imaging at the ascending aorta allowing cardiac output, stroke volume, and aorta velocity time integral calculations as we have done previously (Patel et al., 2015; Kasneci et al., 2017). Analyses of short axis views captured at the level of the papillary muscles allowed calculation of the fractional area change (FAC).

### 2.4 Gut microbiome analyses

Fresh fecal pellets were collected on D0, D1, D3, D5 and D7 from all cohorts. Cecum contents were collected at euthanasia on D3 or D7. Cecum contents and fecal pellets were stored at -20 °C until DNA isolation using the QIAmp PowerFecal Pro DNA Kit (Cat. No. 51804-50, QIAGEN) as per manufacturer’s instructions. DNA purification was performed the week before submission for 16S rRNA amplification and sequencing. DNA quantification and purity analyses were performed using a DS-11 Nanodrop™ spectrophotometer (DeNovix, Delaware, USA). The McGill Genome Centre performed 16S rRNA gene amplification, library preparation, and sequencing, using the MiSeq Reagent Kit and the Illumina MiSeq (Illumina, California, USA) and followed their well-established pipeline which included rigorous and robust technical quality control at every step. Included were water-only samples and repetition of some samples on each 96-well plate submitted. Bacterial DNA was amplified using forward primer - 515F (GTGCCAGCMGCCGCGGTAA) and reverse primer - 806R (GGACTACHVGGGTWTCTAAT) to target the V4 region of 16S rRNA.

Paired end, demultiplexed fastq files were obtained from the McGill Genome Centre. The DADA2 software package (v1.16) (Callahan et al., 2016) was used to remove primers from amplicon sequences, apply taxonomic classification and species-level assignment (using Silva 138.1 prokaryotic SSU taxonomic training data (McLaren and Benjamin, 2021)), remove substitution and chimera errors, and ultimately output an amplicon sequence variant (ASV) table with sample-wise abundances and taxonomy. Both sequencing runs were processed separately and their respective ASV tables were merged using DADA2. Taxonomic and functional profiling, data visualization, and significance testing was performed using MicrobiomeAnalyst v2.0 (Lu et al., 2023).

### 2.5 Gut metabolome analyses

Cecum contents from all cohorts were collected at euthanasia and samples kept at -20 °C until analysis. ~50mg/sample from D3 NoSx and MI cohorts only were prepared for untargeted metabolite profiling (Cheng et al., 2020) and passed through a UPLC–MS/MS platform (Q-Exactive Orbitrap mass spectrometer) in positive and negative mode electronspray ionization using a C18 column to gather a comprehensive metabolite dataset (Cheng et al., 2020). MS raw data processing was performed by the spectral processing module in MetaboAnalyst v6.0 to create a peak list table (Pang et al., 2024). Positive and negative ESI modes were processed independently through peak picking, alignment, deconvolution, and compound annotation in Progenesis QI, yielding 360 and 274 annotated compounds, respectively. Following convention, a cutoff identification score of greater than 70% was used to avoid false positives. MetaboAnalyst v6.0 (Pang et al., 2024) was used for statistical analyses, enrichment, pathway analyses and visualization of the annotated metabolites.

### 2.6 Flow cytometry

Following euthanasia, the small intestine, heart, spleen, and left femoral bone marrow were collected and single cell suspensions prepared for flow cytometry analysis of select innate and adaptive immune cells from all cohorts. Flow cytometry was performed as described previously (Kasneci et al., 2017). **Table 1** contains a list of the antibodies used in the flow cytometry panels.

**Table 1:** Antibodies used in flow cytometry.

Antibody	Clone	Supplier
Brilliant Violet 785-conjugated anti-CD45	309f11	BioLegend
Brilliant Violet 650-conjugated anti-MHCII	M5/114.15.2	BD Biosciences
Phycoerythrin/Dazzle 594-conjugated anti-CD64	x54-5/7.1	BioLegend
efluor450-conjugated anti-CD11b	MI/70	eBioscience
Alexa 488-conjugated anti-Ly6G	RB6-8c5	eBioscience
APC-conjugated anti-Ly6C	AL-21	BD Biosciences
FITC-labeled anti-CD38	90	eBioscience
Phycoerythrin-conjugated anti-MerTK	2b10c42	BioLegend
Alexa 488-conjugated anti-CD8β	YTS156-7.7	BioLegend
APC-Cy7-conjugated anti-CD8α	53-6.7	BioLegend
efluor450-conjugated anti-CD3	eBio500A2	eBioscience
Brilliant Ultra Violet 395-conjugated anti-CD4	GK1.5	BD Biosciences
Phycoerythrin/Dazzle 594-conjugated anti-CD19	1D3	BD Biosciences
Phycoerythrin-conjugated anti-TCRγδ	GL3	BD Biosciences
APC-conjugated anti-TCRαβ	H57-597	BD Biosciences
Alexa 488-conjugated anti-CD25	PC61	BioLegend
Phycoerythrin-conjugated anti-FOXP3	MF-14	BioLegend

List of immune cell-specific antibodies along with their linked fluorescent dye, catalog number, and supplier.

#### 2.6.1 Small intestine

The small intestine (from the base of the stomach to the cecum) was removed from the abdominal cavity. Intestinal contents, adipose tissue, and Peyer’s patches were removed. The cleaned tissue was weighed and a portion of the jejunum weighed and selected for flow cytometry. To begin, the tissue was opened longitudinally and chopped into small pieces. Cells were isolated sequentially from the intraepithelial layer (IEL) and then the lamina propria (LP) (Couter and Surana, 2016; Qiu and Sheridan, 2018). Briefly, to isolate cells from the IEL, samples were incubated end-over-end at 37 °C for 20 min in digestion buffer (RPMI, 5% horse serum, 0.1% 1M DTT, 0.2% 0.5M EDTA), filtered through a 40μm strainer (Falcon, Durham, NC), rinsed with cold RPMI media, and centrifuged to collect single cells. To isolate cells from the LP, tissue captured by the 40μm strainer was rinsed with cold PBS to remove EDTA and incubated in a collagenase-based digestion buffer (RPMI, 5% horse serum, 1% collagenase I, 5mM CaCl_2_, 5mM MgCl_2_) end-over-end at 37 °C for 30 min., filtered through a 40μm strainer, rinsed with cold RPMI media and centrifuged. The cell pellets from IEL and LP extractions were resuspended separately in 1ml of cold FACS buffer (PBS, 5% horse serum, 1% penicillin-streptomycin) and kept on ice.

#### 2.6.2 Heart

The whole heart was sliced open, rinsed free of blood, weighed, minced into small pieces, and incubated end-over-end with a collagenase-based digestion buffer (PBS, 1% collagenase I, 5mM CaCl_2_, 5mM MgCl_2_) at 37 °C for 1 hour, followed by filtration using a 100 μm strainer (Falcon, Durham, NC), then 40μm strainer and rinsed with cold RPMI media (RPMI, 5% horse serum, 2.5% penicillin-streptomycin). Single cell pellets were collected by centrifugation at 1500 rpm at 4 °C for 10 minutes. Single cells were resuspended in 1ml cold FACS buffer.

#### 2.6.3 Bone marrow

Bone marrow was flushed from the isolated left femur using a 1ml tuberculin syringe and cold PBS. Bone marrow was crushed through a 100μm strainer, washed with cold RPMI media, and centrifuged as above. Single cells were resuspended in 1ml cold FACS buffer.

#### 2.6.4 Spleen

The spleen was weighed and mid-spleen portions of ~12-15mg selected for flow cytometry. Spleen tissue was crushed through a 100μm strainer, rinsed with cold RPMI media, and centrifuged as above. Single cells were resuspended in 1ml cold FACS buffer.

Isolated cell samples were incubated with Am-Cyan Live/Dead Fixable Dead Cell stain (Molecular Probes, Carlsbad, CA), blocked with 2.4G2 hybridoma (Fc Receptor Block, ATCC: HB-197), incubated with fluorescently labeled antibodies, washed and fixed with 1% paraformaldehyde. To identify Tregs, cells were incubated with FoxP3 transcription factor Fixation/Permeabilization solution (eBioscience, ThermoFisher Scientific, Inc., Waltham, Massachusetts) for 30 minutes followed by staining with anti-FoxP3 antibody, washing, and fixation with 1% paraformaldehyde. 123count e-beads (cat no. 01-1234, Affymetrix) were added before analyses by flow cytometry using an LSR Fortessa Cell Analyzer (BD Biosciences, San Jose, CA) and DIVA software. Data were analyzed using FlowJo software v10.10 (Tree Star Inc., Ashland, OR).

### 2.7 Intestine histology

Portions of the jejunum from all cohorts were fixed with 4% buffered paraformaldehyde at 4 °C for 2–3 days and then transferred to 70% ethanol and stored at 4 °C until processing and embedding in paraffin. Sections 5 µm thick were cut, stained with H&E, and digitally scanned using a Zeiss Mirax Scanner using Mirax Scan 1.12 software and a 20X objective. Images were imported into QuPath v 0.5.1 (Bankhead et al., 2017). Villi length, crypt, and tunica muscularis thicknesses were measured, and epithelial and goblet cell numbers were enumerated using QuPath.

### 2.8 Bioinformatic and statistical analyses

The open-source MicrobiomeAnalyst v2.0 (Lu et al., 2023) and MetaboAnalyst v6.0 (Pang et al., 2024) programs and commercially available GraphPad Prism™ 10.2.3 program were used to perform bioinformatic and statistical analyses and data visualization.

Raw data from 16S rRNA amplification and sequencing were prepared as described above and imported into MicrobiomeAnalyst v2.0, filtered to remove low-variance features (bottom 10% by interquartile range) and low-prevalence features (present in <20% of samples, minimum count of 2) and normalized using total sum scaling. For assessment of alpha diversity, Chao1 and Shannon diversity indices were calculated using feature-level abundance data and Mann-Whitney/Kruskal-Wallis testing was performed for *post-hoc* pairwise comparisons (*p <* 0.05) using MicrobiomeAnalyst v2.0. We assessed beta diversity using feature-level data using the Bray-Curtis Index with pairwise PERMANOVA (*p <* 0.05) and performed Linear Discriminant Analysis (LDA) Effect Size (LEfSe) at the family level, also using MicrobiomeAnalyst v2.0. A default LDA score (log_10_) threshold of 2.0 and FDR-adjusted p-value cutoff of *q* < 0.05 was used for initial assignment of differentially enriched families. We selected an LDA score threshold of 4.0 to identify taxa with the greatest differential abundance. Next, bacterial family relative abundance was calculated for each sample individually and for each experimental group (samples grouped). T-tests and one-way ANOVA were used to identify significant differences in feature relative abundance between groups, using the Benjamini-Hochberg procedure to yield FDR (*q* < 0.05) if required, on MicrobiomeAnalyst v2.0 (Lu et al., 2023). We chose taxa identified as significantly enriched by LEfSe (LDA score≥4.0) for deeper taxonomic (up to species-level) relative abundance profiling, with one-way ANOVA and Benjamini-Hochberg FDR (*q* < 0.05) to determine statistical significance between groups. The LEfSe-based cladogram was prepared using Galaxy, an open-source platform (Community TG, 2024) available at https://galaxyproject.org and hosted by the Metabiome Portal at George Mason University.

To assess the relationship between post-MI gut microbial community shifts and cecum metabolite changes, we performed integrative microbiome-metabolome analysis using the Microbiome Metabolomics Profiling (MMP) module of MicrobiomeAnalyst 2.0 (Lu et al., 2023). Paired 16S rRNA amplicon sequencing and untargeted metabolomics data from MI D3 and NoSx cecum samples (n=7 per group, both sexes pooled) were used as input. For the microbiome data, OTU-level counts were prepared as above and were then aggregated at the family level. For the metabolomics data, positive and negative electronspray ionization modes were annotated as above and then merged into a single list of 626 unique metabolites; 8 compounds detected in both polarities were resolved by retaining the mode with higher between-group variance (F-ratio; 6 kept from positive, 2 from negative). Metabolite data were log_2_-transformed prior to upload, then normalized by median and auto-scaled (mean-centered and divided by standard deviation of each feature) within MicrobiomeAnalyst 2.0.

Distance correlation analysis (Székely et al., 2007) was performed between family-level microbial abundances and annotated metabolites using the MMP module. Distance correlation captures both linear and non-linear associations and produces a non-negative coefficient (dCor) ranging from 0 (no association) to 1 (perfect dependence). Associations with dCor > 0.5 and *p* < 0.05 were considered significant. Results were visualized as a hierarchically clustered heatmap.

Pathway enrichment analysis was performed within the MMP module against the predicted metabolic capacity of the detected microbial taxa using the KEGG pathway database (Mus musculus) and the full set of unidentified LC-MS peaks (27,432 features) gathered from the positive and negative mode. This approach contextualizes metabolite changes within the metabolic potential of the observed microbial community, rather than relying on generic pathway databases. Enriched pathways with Gamma-adjusted *p* < 0.05 were selected as significant.

Regarding data visualization, we imported data gathered from microbiome, metabolome, immune profiling, histological analyses or echocardiography into GraphPad Prism™ 10.6. Outliers were removed using Robust Nonlinear Regression and Outlier Detection (ROUT) and the default Q value of 1%. Significance testing used ANOVA and correction for multiple comparisons with Tukey *post-hoc* tests. Box plots display each sample’s data point along with the mean and standard deviation in our figures.

## 3 Results

### 3.1 Sex-specific gut microbiota profiles at baseline in retired breeder C57BL/6N mice

**Figure 1:**
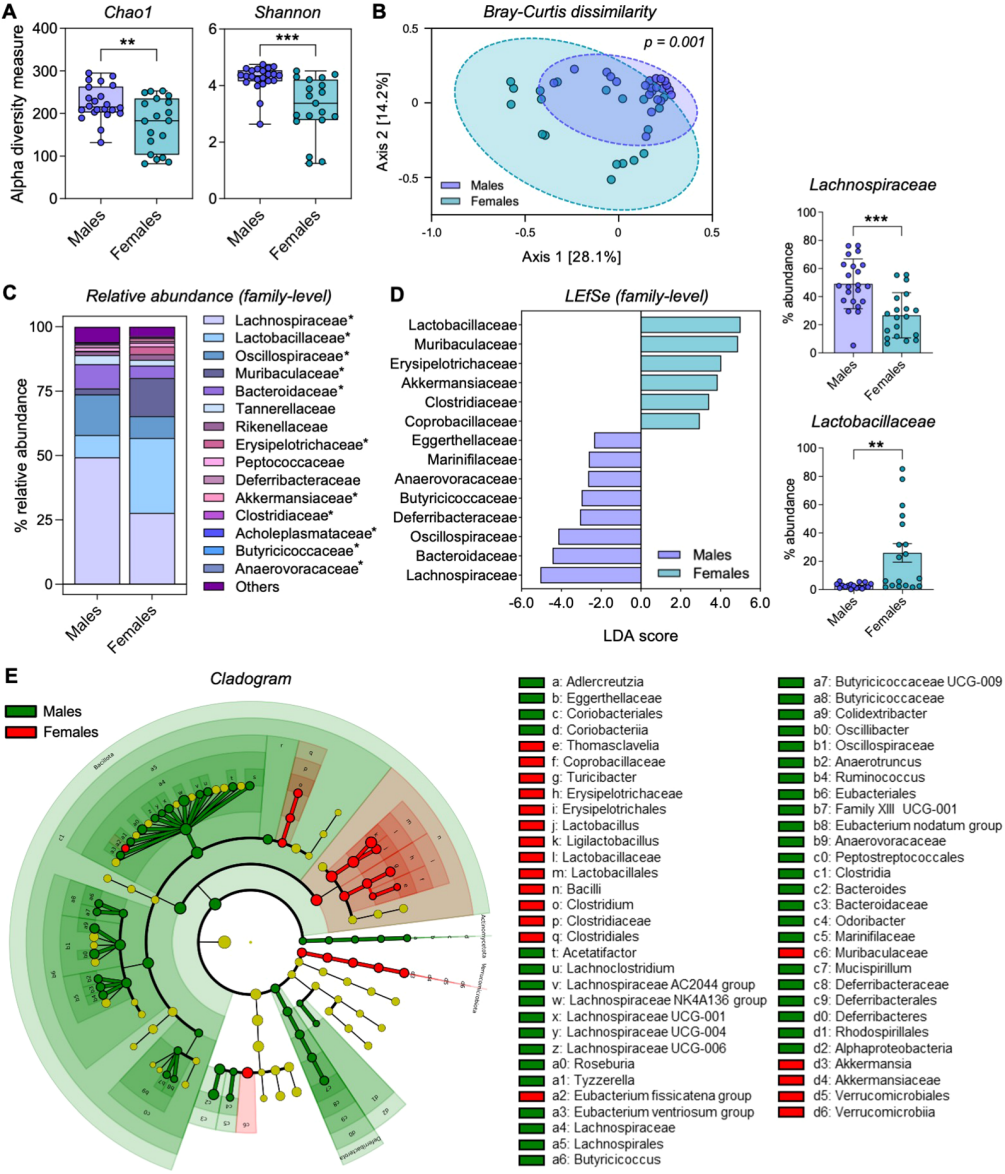
Microbiota composition at baseline. Fecal pellets from retired breeder mice (n=19 females, n=22 males) were collected after 4 weeks of acclimation to the vivarium and prior to surgery to analyze baseline gut microbiota composition. 16S rRNA sequencing of fecal DNA enabled microbiota profiling. Analyses were performed using MicrobiomeAnalyst v2.0. LEfSe-based cladogram was prepared using Galaxy, an open-source platform hosted by George Mason University. **(A)** Differences in alpha (within-sample) diversity between male and female microbiota. Chao1 and Shannon indices of alpha diversity reflect taxonomic richness (Chao1 and Shannon) and evenness (Shannon) of microbiota. **p < 0.01, ***p < 0.001. **(B)** Bray-Curtis Index with PERMANOVA was used to calculate beta (between-sample) diversity in microbiota samples collected from males and females at baseline. Results are visualized by spatial clustering on PCoA plots. **(C)** Relative abundance of the 15 most abundant bacterial families at baseline in males and females. * indicates significant differential abundance (unpaired Student’s t-test with Benjamini-Hochberg FDR correction, *q* < 0.05). **(D)** Left: Microbial features differentially enriched in male and female microbiota identified by Linear discriminant analysis Effect Size (LEfSe). LDA scores represent the effect size of microbial features. Only taxa with an LDA score >2 and *q* < 0.05 were considered. Right: Percent relative abundance of taxa with the highest LDA scores from males and females. Significance calculated using an unpaired Student’s t-test. **p < 0.01, ***p < 0.001. **(E)** LEfSe-derived cladogram of discriminative bacterial taxa between males and females at baseline.

To identify whether sex-specific differences in the gut microbiome were present at baseline, we characterized gut microbiota composition of retired breeder male and female mice before surgery, **Figure 1**. Feature-level alpha diversity, assessed using Chao1 (richness; number of observed taxa) and Shannon (richness and evenness; extent of even distribution of different taxa) indices, was significantly lower in females than males, indicating reduced microbial richness and evenness in female gut microbiota, **Figure 1A**. Further, assessment of beta diversity using Bray-Curtis dissimilarity showed significant differences in community composition between male and female samples (PERMANOVA, *p* = 0.001), as visualized by PCoA, **Figure 1B**.

To characterize these sex-associated microbiota differences at baseline, relative abundance profiling was performed at the family level. The top 15 most abundant bacterial families are shown in **Figure 1C**. In total, 9 bacterial families were significantly differentially abundant with sex (unpaired Student’s t-test with Benjamini-Hochberg [FDR], *q* < 0.05), with 7 present in the top 15. Notably, the top 5 families made up ~80% of the total abundance. Using our LEfSe results, we selected an LDA score of greater than 4.0 to detect the most relevant, consistently differential taxa between males and females at baseline, **Figure 1D**. At the family level, *Lactobacillaceae, Muribaculaceae*, and *Erysipelotrichaceae* were significantly enriched in female microbiota, while *Lachnospiraceae, Bacteroidaceae*, and *Oscillospiraceae* were enriched in the males. Among the most differentially abundant families, *Lactobacillaceae* relative abundance was 3-fold higher in females than males, whereas females had ~50% of the *Lachnospiraceae* relative abundance observed in males. To further explore these sex-specific differences, a LEfSe-derived cladogram depicting the hierarchical structure of differentially enriched bacterial lineages was created, **Figure 1E**. We conclude that the gut microbiota composition differs by sex at baseline in these single-sourced, retired breeder C57BL/6N mice. Given these baseline differences, subsequent analyses were performed separately in male and female cohorts.

### 3.2 Distinct physiological responses after SH and MI

To investigate physiological changes during the surgical recovery period, we evaluated body weight, organ weights, and changes to cardiac structure and function with time after MI-inducing (MI) versus sham (SH) surgery. The surgeries share anesthesia, analgesia, intubation, and thoracotomy, but only MI induces significant cardiac tissue damage. Significant reductions in body weight from the day of surgery (D0/NoSx) to day 3 (D3) post-surgery were found in males and females after both SH and MI, **Supplementary Figures 1A, B**. In males, body weight remained significantly reduced until D7. In contrast, females regained body weight by D7, approaching healthy NoSx body weight values.

To begin investigation of the post-surgical intestinal milieu, cecum contents from males and females at euthanasia were collected, weighed, and content weight indexed to body weight, **Supplementary Figure 1C**. After MI, cecum weight was significantly reduced on D3 in males and females, but comparable to NoSx controls by D7. In contrast, cecum weight was unaffected after SH in males on D3 and increased on D7, when compared to NoSx controls. In females, cecum weight remained comparable between SH and NoSx cohorts and thus was not affected by SH. Overall, we found that MI, but not SH, induced early, acute reductions in the cecum content weight in both sexes.

Lastly, to validate the MI model and to measure changes to left ventricle (LV) cardiac structure and function after MI over time, we performed echocardiography on D3 and D7, prior to euthanasia, **Table 2**. As expected, no changes in LV structure or systolic function were found in SH males or females when compared with their NoSx cohorts. In males, MI induced expected increases in LV volume and area (measure of structure) on D3 and D7, indicating significant, lasting LV dilation. In females, MI-induced increases in LV volume and area were delayed and only evident on D7. Despite similar, lasting reductions in LV fractional area change (FAC; measure of function) after MI in males and females on D3, no changes in measure of LV systolic output such as LV cardiac output, LV stroke volume, or aortic velocity-time integral (Ao VTI) were detected. These data demonstrate delayed LV dilation in female mice post-MI and a preservation of global systolic function despite loss of some LV contractional function, in both sexes.

**Table 2:** Echocardiography measures of cardiac structure and function in male and female mice.

Intervention	Male					Female				
	NoSx	SH		MI		NoSx	SH		MI	
*Day (n)*	(18)	*D3 (n)*	*D7 (n)*	*D3 (n)*	*D7 (n)*	(18)	*D3 (n)*	*D7 (n)*	*D3 (n)*	*D7 (n)*
LV Vol (d) (ml)	69.3 ± 4.0	59.8 ± 11.9	82.1 ± 4.4	103.4 ± 36.7*†	105.6 ± 13.3*	70.6 ± 2.0	62.5 ± 11.6	83.1 ± 3.2	68.8 ± 14.7⁋	107.1 ± 6.4*†‡
LV Vol (s) (ml)	36.4 ± 3.4	28.4 ± 11.6	48.4 ± 3.4	70.7 ± 29.4*†	77.5 ± 14*	43.2 ± 2.0	37.8 ± 11.1*	53.4 ± 2.7	45.9 ± 17.2	80.4 ± 4.0*†‡
LV area (d) (mm^2^)	25.0 ± 0.9	22.5 ± 2.9	27.3 ± 1.0	30.4 ± 6.4*†	31.3 ± 2.2*	25.2 ± 0.5	23.6 ± 2.9	27.9 ± 0.7	24.2 ± 3.2⁋	32.4 ± 1.1*†‡
LV area (s) (mm^2^)	16.5 ± 1.0	14.3 ± 3.6	19.7 ± 0.9	24.0 ± 6.1*†	25.7 ± 2.7*	18.6 ± 0.5	16.8 ± 3.7	21.1 ± 0.8	18.6 ± 4.1	27.4 ± 1.1*†‡
FAC (%)	53.5 ± 3.1	52.4 ± 8.0	43.1 ± 2.6	24.8 ± 16*†	22.9 ± 3.7*	41.2 ± 2.2⁋	45.0 ± 8.9	34.5 ± 2.7	23.9 ± 11.7*	30.3 ± 2.7*
CO (mL/min.)	46.6 ± 3.5	42.7 ± 10.4	51.5 ± 4.0	38.0 ± 6.0	37.8 ± 2.0	40.6 ± 3.5	37.8 ± 12	42.4 ± 1.9	41.9 ± 7.1	33.5 ± 1.9
SV (ml)	89.0 ± 7.2	81.4 ± 18	97.9 ± 7.5	74.7 ± 1.4	72.7 ± 4.0	77.7 ± 6.5	70.2 ± 22	80.9 ± 3.4	81.2 ± 13.1	65.3 ± 3.6
Ao VTI (mm)	49.8 ± 2.8	46.5 ± 9.5	53.2 ± 2.5	42.0 ± 6.3	42.9 ± 2.0	41.1 ± 2.5⁋	40.2 ± 7.8	40.2 ± 1.4	39.1 ± 8.5	34.4 ± 1.7

Echocardiography was performed in NoSx mice and SH and MI mice 3 days (D3) or 7 days (D7) after surgery. The LV internal volume and area in systole and diastole, and LV fractional area change were calculated from EKV-gated acquisitions of the long axis view and short axis view, respectively. Ao VTI, cardiac output, and stroke volume were calculated from pulsed wave Doppler images of the ascending aorta. Significance was determined using ANOVA with Tukey’s *post-hoc* testing (*p <* 0.05). Statistical significance is indicated by an * in comparison with NoSx, † in comparison with SH, ‡ in comparison with D3, and ⁋ in comparison with opposite sex.

### 3.3 Surgery-specific gut microbiota changes in males after SH and MI

**Figure 2:**
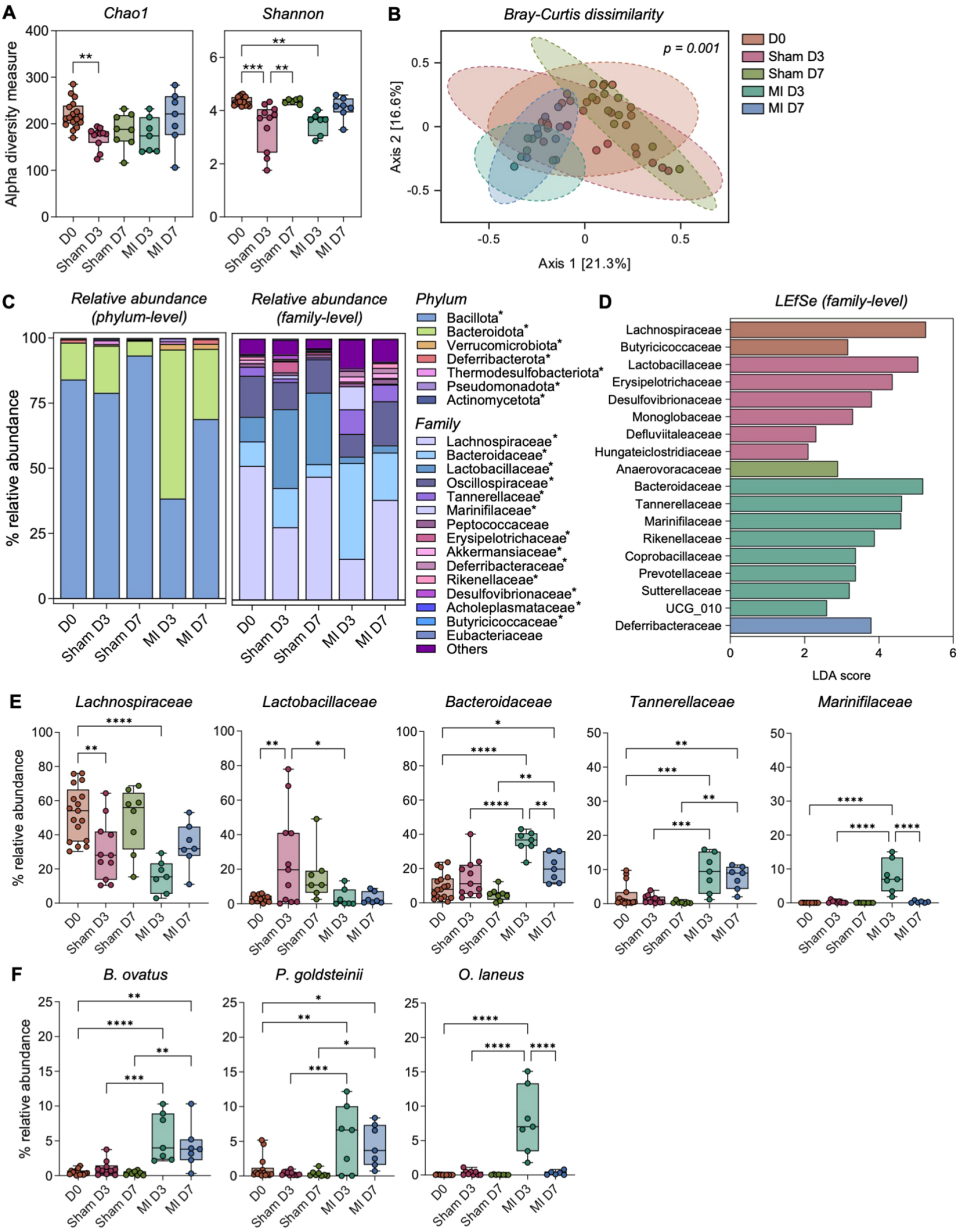
Microbiota composition at different timepoints after SH and MI in males. Fecal pellets were collected from male mice on the day of surgery (D0) and day 3 (D3) or day 7 (D7) after SH or MI. 16S rRNA sequencing of fecal DNA enabled microbiota profiling. Analyses were performed using MicrobiomeAnalyst v2.0. Each dot indicates a sample from an individual mouse. **(A)** Differences in alpha (within-sample) diversity after SH and MI at different timepoints. Chao1 and Shannon indices of alpha diversity reflect taxonomic richness (Chao1 and Shannon) and evenness (Shannon) of microbiota. **p < 0.01, ***p < 0.001. **(B)** Bray-Curtis Index with PERMANOVA was used to calculate beta (between-sample) diversity in microbiota samples collected at different timepoints after SH and MI. Results are visualized by spatial clustering on PCoA plots. **(C)** Changes in percent relative abundance of the 15 most abundant bacterial phyla and families after SH or MI. * indicates significantly differential abundance (one-way ANOVA with Benjamini-Hochberg FDR correction, *q* < 0.05). **(D)** LEfSe showing most differentially abundant taxa in male microbiota at different timepoints after SH and MI (LDA score >2, *q* < 0.05). LDA scores represent the effect size of each taxon. **(E, F)** Relative abundance of the discriminative taxa found in MI D3 samples at the family level **(E)** and species level **(F)**. Significance was calculated using ANOVA with Tukey’s *post-hoc* test. *p < 0.05, **p < 0.01, ***p < 0.001, ****p < 0.0001.

To determine the impact of MI on gut microbiota over time in retired breeder males, we compared gut microbial profiles of fecal pellets collected on D1, D3, D5, and D7 after MI with those collected on D0, **Supplementary Figure 2**. Significant reductions in Shannon alpha diversity were found on D3 and D5 after MI, **Supplementary Figure 2A**. However, no differences in alpha diversity using the Chao1 diversity index were found. PCoA plots of Bray-Curtis dissimilarity revealed significant differences in bacterial community composition between samples collected on D0 and all other time points, as well as between samples from D3 and D7, **Supplementary Figure 2B**. A heatmap shows the relative abundances of identified bacterial families, **Supplementary Figure 2C**. With a threshold of LEfSe LDA score ≥ 4.0 and percent relative abundance greater than 1%, we identified taxa that varied significantly in abundance with time after surgery. Specifically, we detected time-dependent expansions in *Bacteroidaceae*, *Rikenellaceae*, and *Marinifilaceae*, alongside decreases in *Oscillospiraceae* and *Lachnospiraceae* which peaked on D3 and did not reach baseline levels by D7. In particular, *Rikenellaceae* and *Marinifilaceae* showed sharp, acute spikes in relative abundance on D1 and D3, respectively. Ultimately, as depicted in our temporal abundance profiling, the most dramatic changes to microbial populations were found on D3 after surgery. Thus, subsequent analyses of the acute impact of SH and MI on the gut microbiota focused on D3 and D7 timepoints, comparing fecal samples collected on D0, D3 and D7, **Figure 2**.

Significant changes in alpha diversity were found using Chao 1 and Shannon diversity indices, **Figure 2A**. Specifically, Chao1 alpha diversity was decreased on D3 after SH surgery only, while Shannon alpha diversity was decreased on D3 after both SH and MI surgeries when compared with D0 samples. Measures of Bray-Curtis dissimilarity revealed significant differences in gut microbiota community composition between D0 samples and all other experimental groups, indicating an impact of surgery on gut microbiota, regardless of surgery type, that was not resolved by D7, **Figure 2B**. Among the surgical groups, while MI D3 showed significant differences in community composition when compared to its D7 counterpart, *p* = 0.027, there was no significant difference detected when SH D3 was compared to SH D7. However, SH samples showed significant Bray-Curtis dissimilarity compared to their MI counterparts on D3, *p* = 0.001, and D7, *p* = 0.0001. Together, these data indicate surgery-specific and time-dependent changes in gut microbiota in male mice.

At the phylum level, relative abundance profiling (one-way ANOVA with Benjamini-Hochberg FDR, *q* < 0.05) revealed significant expansions of *Bacteroidota*, *Pseudomonadota (Proteobacteria)*, *Verrucomicrobiota*, and *Thermodesulfobacteriota (Desulfobacterota)* on D3 after MI, alongside reductions in *Bacillota (Firmicutes)* and *Deferribacterota*, **Figure 2C**. SH mice showed increases in *Pseudomonadota* and *Thermodesulfobacteriota* populations on D3, with decreases in *Deferribacterota* and *Bacillota (Firmicutes).* Together these data suggest development of surgery-associated microbial patterns. At the family level, 13 of the top 15 most abundant taxa showed significant changes in relative abundance after surgery, with the most drastic microbiota restructuring found in MI mice on D3, **Figure 2C**. To identify the most strongly differentiated families, LEfSe analysis was performed, **Figure 2D**. Specifically, D3 samples showed the greatest number of significant, distinguishing microbial associations, with more taxa identified in MI than SH samples. To identify the most discriminative taxa, LEfSe LDA score ≥ 4 and percent abundance greater than 1% were chosen for further relative abundance profiling, **Figure 2E**.

D0 microbiotas were most enriched in *Lachnospiraceae*, with a significant decline in relative abundance in SH D3 and MI D3 samples, consistent with a reduction following surgery. SH D3 mice were associated with reduced *Lachnospiraceae* and increased *Lactobacillaceae* but were otherwise indistinguishable from D0 samples. Importantly, more abundant and significant gut microbiota alterations were found in MI versus SH mice. MI D3 had the greatest differential abundance of *Bacteroidaceae, Tannerellaceae*, and *Marinifilaceae* taxa when compared with other cohorts. The sharp accumulation of *Marinifilaceae* in MI D3 appeared acute, while increased relative abundance of *Bacteroidaceae* and *Tannerellaceae* was present in MI D7 as well. There were no microbial families with an LDA score greater than 4.0 in SH D7 or MI D7 mice. Further, when probing for relative abundance at the species level, *Bacteroides ovatus* (*Bacteroidaceae), Odoribacter laneus* (*Marinifilaceae)*, and *Parabacteroides goldsteinii* (*Tannerellaceae)* were found significantly increased on D3 and D7 after MI but were not changed in SH samples at any timepoint, **Figure 2F**.

Overall, we identified distinct surgery-specific and time-dependent differences in several microbial populations. Male mice showed dynamic changes to their gut microbial composition, particularly on D3 following surgery, with the greatest microbial remodeling found in MI mice. Regarding temporal changes, gut microbiota did not fully resemble baseline composition by D7.

### 3.4 Surgery-specific gut microbiota changes in females after SH and MI

Next, the temporal effects of MI on gut microbiota were investigated in females, using fecal pellets collected on D0, D1, D3, D5, and D7 in mice after MI, **Supplementary Figure 3**. No significant changes in alpha diversity were found, at any time point, except for a small but significant increase in Chao1 alpha diversity on D1, **Supplementary Figure 3A**. PCoA plots of Bray-Curtis dissimilarity revealed significant differences in microbiota composition with time, when D0 was compared to D1, D3 and D5, **Supplementary Figure 3B**. A heatmap was created to temporally visualize relative abundances of identified bacterial families, **Supplementary Figure 3C**. Using LEfSe and abundance profiling at the family level, we identified time-dependent expansions of *Bacteroidaceae*, *Tannerellaceae*, *Marinifilaceae*, *Rikenellaceae*, and *Eubacteriaceae* communities, with peaks on either D1 or D3 after surgery. Overall, time-dependent changes in relative abundance were present in females after MI surgery, which were different from those detected in males. We also found that, as in males, D1 and D3 showed the most drastic differences in microbial populations, with only partial resemblance to D0 profiles by D7.

**Figure 3:**
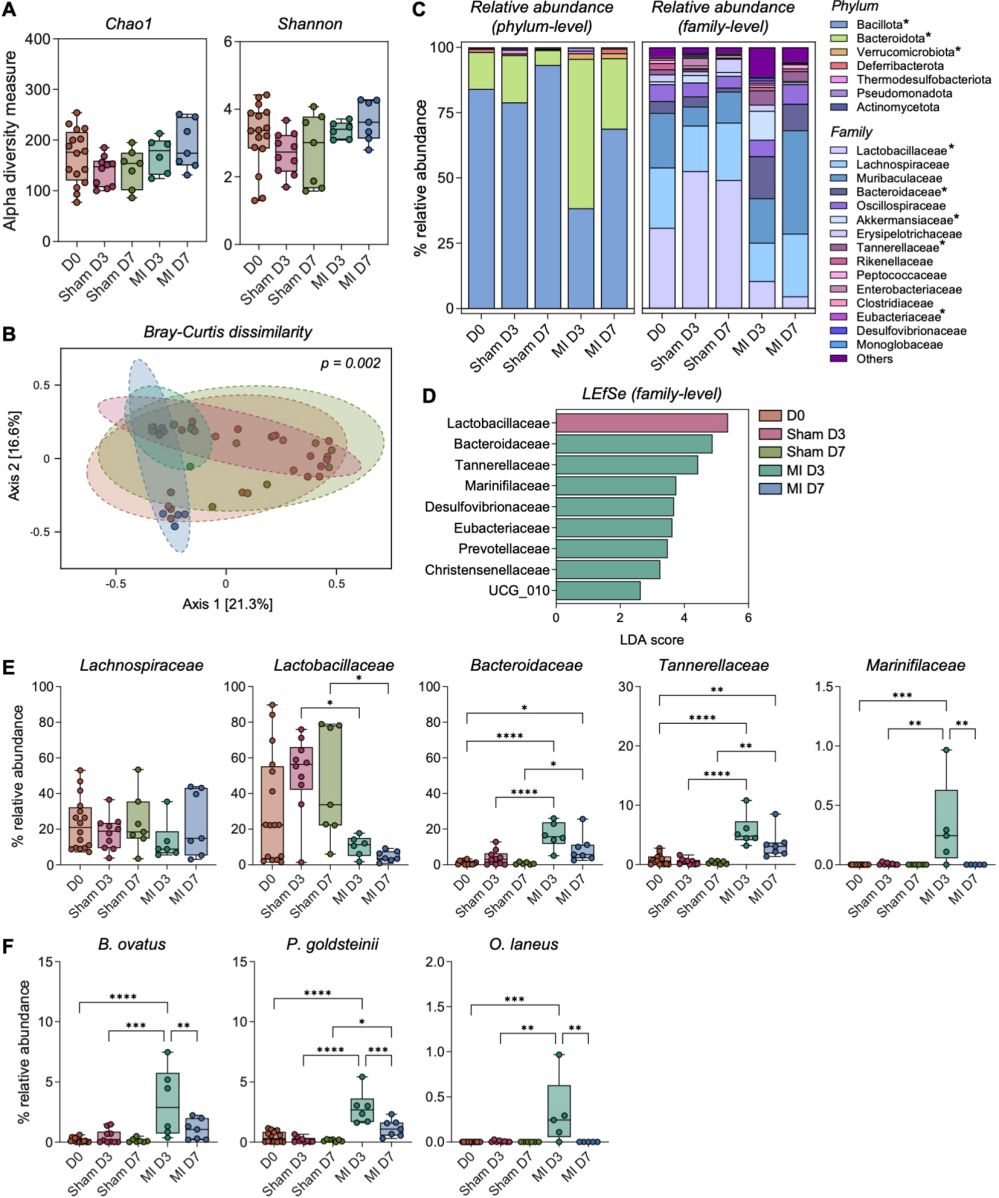
Microbiota composition at different timepoints after SH and MI in females. Fecal pellets were collected from female mice on the day of surgery (D0) and day 3 (D3) or day 7 (D7) after SH or MI. 16S rRNA sequencing of fecal DNA enabled microbiota profiling. Analyses were performed using MicrobiomeAnalyst v2.0. Each dot indicates a sample from an individual mouse. **(A)** Differences in alpha (within-sample) diversity after SH and MI at different timepoints. Chao1 and Shannon indices of alpha diversity reflect taxonomic richness (Chao1 and Shannon) and evenness (Shannon) of microbiota. **(B)** Bray-Curtis Index with PERMANOVA was used to calculate beta (between-sample) diversity in microbiota samples collected at different timepoints after SH and MI. Results are visualized by spatial clustering on PCoA plots. **(C)** Changes in percent relative abundance of the 15 most abundant bacterial phyla and families after SH or MI. * indicates significantly differential abundance (one-way ANOVA with Benjamini-Hochberg FDR correction, *q* < 0.05). **(D)** LEfSe showing most differentially abundant taxa in female microbiota at different timepoints after SH and MI (LDA score >2, *q* < 0.05). LDA scores represent the effect size of each taxon. **(E, F)** Relative abundance of the discriminative taxa found in MI D3 samples at the family level **(E)** and species level **(F)**. Significance was calculated using ANOVA with Tukey’s *post-hoc* test. *p < 0.05, **p < 0.01, ***p < 0.001, ****p < 0.0001.

Focusing on D3 and D7 timepoints, the impact of MI and SH on gut microbiota in females was more deeply investigated using fecal pellets collected on D0, D3, and D7, **Figure 3**. Unlike the male cohorts, no significant changes in alpha diversity were identified in females, **Figure 3A**. Beta diversity analysis using Bray-Curtis dissimilarity revealed significant differential gut microbiota composition between D0 and MI D3, *p* = 0.006, but not between D0 and any SH or MI D7 samples. However, MI D3 samples demonstrated differential microbiota composition relative to MI D7, *p* = 0.045, and SH D3, *p* = 0.008, **Figure 3B**.

At the phylum level, relative abundance profiling (one-way ANOVA with Benjamini-Hochberg FDR, *q* < 0.05) revealed reductions in *Bacillota (Firmicutes)* and *Deferribacterota*, alongside expansions of *Bacteroidota, Verrucomicrobiota*, and *Thermodesulfobacteriota* populations in MI samples only, with the greatest changes found on D3, **Figure 3C**. At the family level, 5 of the top 15 most abundant families differed significantly in relative abundance after MI, **Figure 3C**. LEfSe analyses revealed a single significantly differential taxon in SH samples, and multiple significantly enriched taxa in MI samples, **Figure 3D**. After SH surgery, a differential relative abundance of *Lactobacillaceae* was detected. In contrast, increased relative abundance of *Bacteroidaceae, Tannerellaceae, Marinifilaceae, Desulfovibrionaceae, Eubacteriaceae, Prevotellaceae, Christensenellaceae*, and *UCG_010* were identified on D3 in MI females. LEfSe analyses did not uncover specific bacterial enrichment in D0, SH D7, or MI D7 samples. A cut-off of LDA score ≥ 4.0 was used to highlight the top discriminative taxa for further abundance profiling.

Unlike the males, *Lachnospiraceae* did not vary with surgery in females, **Figure 3E**. Rather, the relative abundance of *Lactobacillaceae* was reduced in MI, whereas *Bacteroidaceae* and *Tannerellaceae* were increased in MI when compared to their D0 and SH counterparts. Like the males, *Marinifilaceae* was increased on MI D3 only, while the increased relative abundances of *Bacteroidaceae* and *Tannerellaceae* detected on MI D3 were also found in MI D7. No significant changes in any of these taxa were identified in SH samples, **Figure 3E**. To further explore these significant bacterial families, we investigated relative abundance at the species level, **Figure 3F**. We found that *Bacteroides dorei* (*Bacteroidaceae)*, *Bacteroides ovatus* (*Bacteroidaceae)*, and *Parabacteroides goldsteinii* (*Tannerellaceae)* were increased in female MI D3 and MI D7 samples, while *Odoribacter laneus (Marinifilaceae)* was increased only in MI D3 samples. Comparisons between SH and D0 samples showed no difference in relative abundance in any of these taxa, **Figure 3F**. Hence, unlike in the males, significant changes to gut microbiota composition in females appeared only after MI and not after SH, peaking on D3 with a return toward baseline microbiota composition by D7.

### 3.5 Peak sex-specific gut microbiota differences on D3 after MI

**Figure 4:**
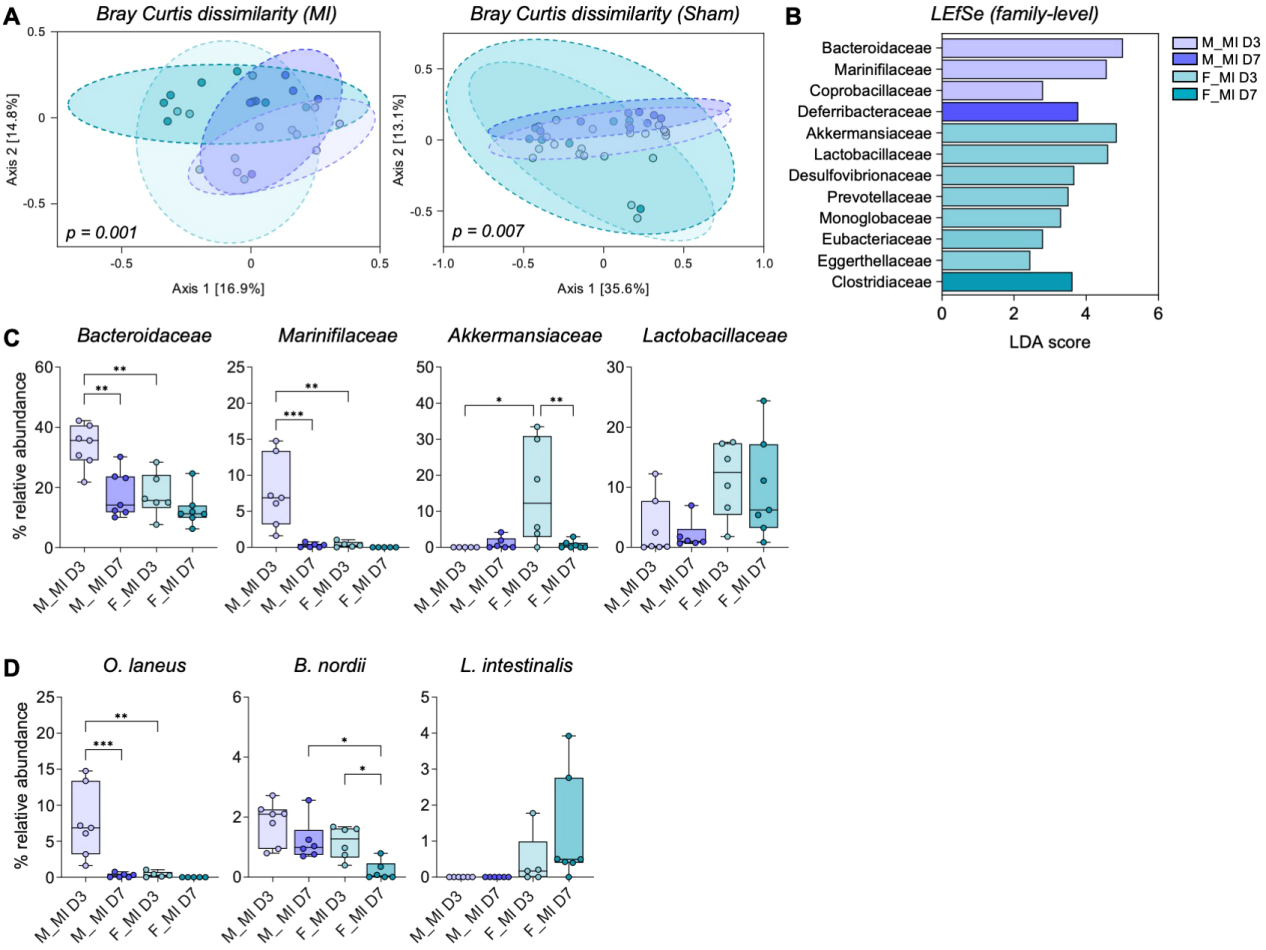
Sex-specific changes in microbiota after SH and MI. Fecal pellets were collected from male and female mice 3 days (D3) or 7 days (D7) after SH or MI. 16S rRNA sequencing of fecal DNA enabled microbiota profiling. Analyses were performed using MicrobiomeAnalyst v2.0. Each dot indicates a sample from an individual mouse. **(A)** Bray-Curtis Index with PERMANOVA was used to calculate beta (between-sample) diversity in microbiota samples collected at different timepoints after SH and MI from males and females. Results are visualized by spatial clustering on PCoA plots. **(B)** LEfSe showing most differentially abundant taxa in male and female microbiota at different timepoints after SH and MI (LDA score >2, *q* < 0.05). LDA scores represent the effect size of each taxon. **(C, D)** Relative abundance of the most discriminative bacterial families **(C)** and species **(D)** from male and female MI D3 samples. Significance was calculated using ANOVA with Tukey’s *post-hoc* test. *p < 0.05, **p < 0.01, ***p < 0.001.

To explicitly evaluate the impact of biological sex on gut microbiota compositional changes after SH and MI, we compared gut microbiota on D3 and D7 between males and females, **Figure 4**. Chao1 and Shannon indices of alpha diversity revealed no significant sex-specific differences in alpha diversity. Conversely, Bray-Curtis dissimilarity assessment of beta diversity revealed significant differences in community composition between female MI D3 and male MI D3 samples, *p* = 0.009, but not between female MI D7 and male MI D7 samples, *p* = 0.06, as visualized by PCoA, **Figure 4A**. Within each sex, differential bacterial communities were also significantly different between MI D3 and MI D7 in females, *p* = 0.024, and males, *p* = 0.024. No differences were identified within SH groups.

Later, LEfSe identified the greatest number of differentially abundant taxa on D3 after MI, with fewer on D7, **Figure 4B**. Focusing on taxa with an LDA score ≥ 4.0, relative abundance profiling revealed increased *Bacteroidaceae* and *Marinifilaceae* in males after MI on D3, while a greater abundance of *Akkermansiaceae* and *Lactobacillaceae* were found in MI D3 females, **Figure 4C**. Together, these data highlight sex-specific differences in taxonomic relative abundance after MI. When probed at the species level, the relative abundances of *Odoribacter laneus* (*Marinifilaceae)* was greater in male MI D3 samples, *Bacteroides nordii* (*Bacteroidaceae)* was lowest in females on MI D7, and *Akkermansia muciniphila (Akkermansiaceae)* was highest in female MI D3 samples, **Figure 4D**. Despite significant LEfSe results, the relative abundances of *Lactobacillaceae*, **Figure 4C**, and its species *Lactobacillus intestinalis*, **Figure 4D**, were not significantly following ANOVA with *post-hoc* correction. Again, no significant differences in taxonomic relative abundance were detected after SH surgery. Overall, these data suggest that sex-specific differences in gut microbial relative abundance were most evident on D3 after MI.

### 3.6 Sex-specific metabolomic and microbe-metabolite signatures on D3 after MI

**Figure 5:**
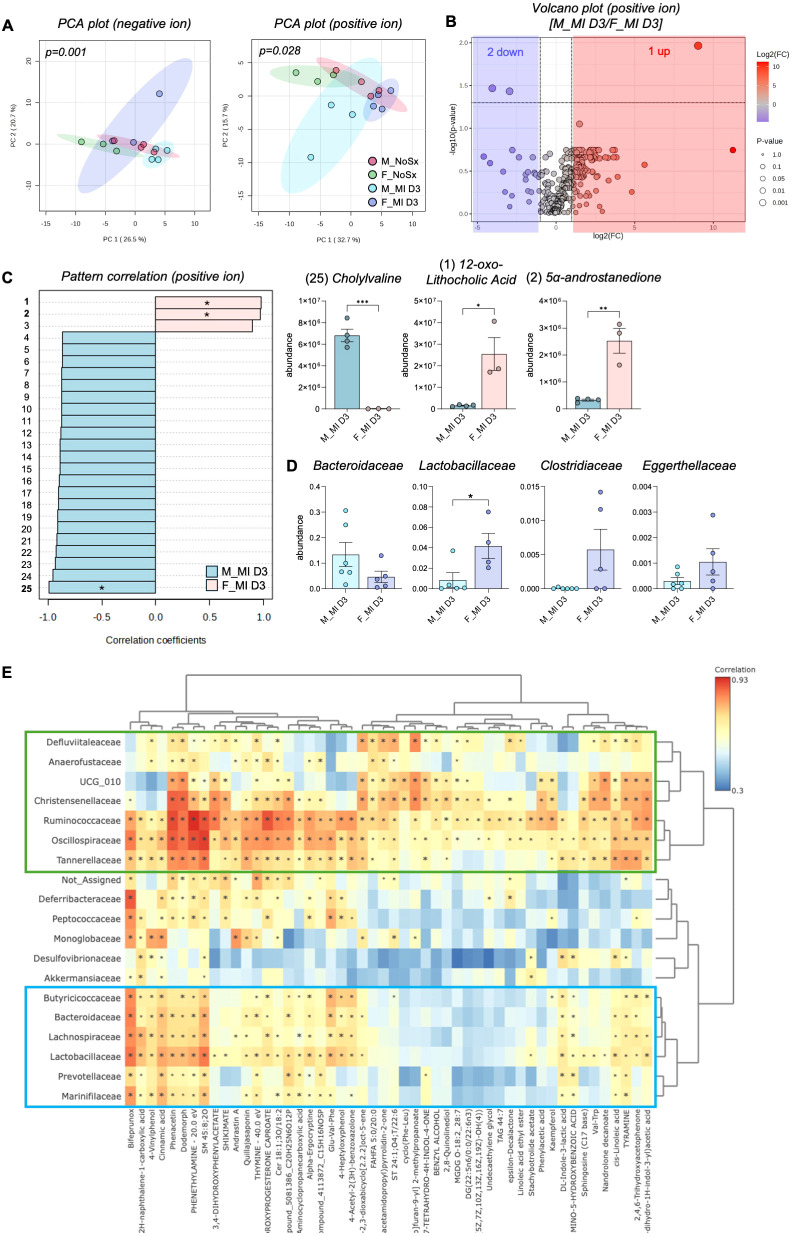
Gut metabolite profiles and microbe-metabolite associations after MI. Cecum contents were collected from NoSx or MI mice on D3. Untargeted UPLC-MS in the negative and positive ion modes allowed for metabolite quantification and characterization. 16S rRNA sequencing of cecal DNA enabled microbiota profiling. Analyses were performed using MetaboAnalyst v6.0 and MicrobiomeAnalyst v2.0. Each dot indicates a sample from an individual mouse. **(A)** Principal Component Analysis (PCA) of both negative and positive ion mode metabolites shows significant differences in metabolite profiles between NoSx and MI D3 cecal metabolomes in males and females (p-values calculated with PERMANOVA). Results are visualized on PCA plots. **(B)** Volcano plot shows significant changes in male MI D3/female MI D3 metabolite abundance (log_2_ fold change [log_2_FC]; significance determined using an empirical Bayes-moderated statistic) in the positive ion mode, revealing 2 upregulated (increased) metabolites and 1 downregulated (decreased) metabolite in male over female samples (adjusted *p*-value < 0.05). **(C)** Left: Pearson r correlation analyses of positive ion mode metabolites showing male-female pattern correlation (top 25 metabolites) from MI D3 cecal sample. * indicates significantly differential abundance (one-way ANOVA with Benjamini-Hochberg FDR correction, *q* < 0.05). Right: Relative abundance of cholylvaline, 12-oxo-lithocholic acid, and 5a-androstanedione. Significance determined by unpaired Student’s t-test, *p <* 0.05. **(D)** Relative abundance of bacterial families involved in secondary bile acid metabolism and amino acid conjugation. Significance determined by unpaired Student’s t-test. *p < 0.05, **p < 0.01, ***p < 0.001. **(E)** Microbiota-metabolome distance correlation heatmap comparing MI D3 and NoSx groups. Correlation between family-level microbial abundances and annotated cecum metabolites (626 compounds) in MI D3 (n=7) and NoSx (n=7) samples. Rows are bacterial families (family order fixed to emphasize guild structure); columns are metabolites clustered hierarchically. * denotes significant associations (dCor > 0.5, *p* < 0.05); color scale spans dCor = 0.3 (blue) to 0.93 (red). Three vertically stacked blocks are evident: a top block of seven homeostatic fiber-fermenting families; a bottom block of six MI-expanded families; and a middle block of five families. Microbiome data were TSS-normalized; metabolite data were log_2_-transformed, median-normalized, and auto-scaled within the MMP module of MicrobiomeAnalyst 2.0.

To characterize the dynamics of microbial-associated metabolites accompanying changes to microbiota populations in the gut, cecum samples from NoSx and MI D3 cohorts were collected and metabolites analyzed using untargeted metabolomics. A total of 360 and 274 metabolites were identified in the positive and negative modes, respectively. PCA plots for both negative and positive ion modes show significant differences in metabolite profiles between NoSx and MI D3 groups in males and females (p-values calculated with PERMANOVA), **Figure 5A**. No significant differences were found when NoSx male and female mice were compared, **Figure 5A**. Investigating specific metabolite differences after MI, a volcano plot shows significant changes in metabolite abundance (log_2_ fold change [log_2_FC] derived from a limma linear model fitted to log_2_-transformed data; significance determined using an empirical Bayes-moderated statistic) in the positive ion mode, revealing 2 upregulated (increased) metabolites and 1 downregulated (decreased) metabolite in male over female samples, **Figure 5B**. No significant differences were found in metabolites using the negative ion mode. Further, Pearson r correlation analyses of positive ion mode metabolites showing male-female pattern correlation from MI D3 samples revealed significantly higher relative abundance of cholylvaline in males and higher relative abundance of 12-oxo-lithocholic acid and 5α-androstanedione in females, **Figure 5C** and **Table 3**. Cholylvaline and 12-oxo-lithocholic are gut bacterial metabolites derived from liver-produced deoxycholic acid and chenodeoxycholic acid, respectively (Mohanty et al., 2024). No significant differences in precursor deoxycholic acid or chenodeoxycholic acid levels were identified.

**Table 3:** Male-female pattern correlation of positive ion mode metabolites from MI D3 samples.

Compound number	Compound name	Correlation coefficient
1*	(4R)-4-((3S,5S,7S,9S,10S,13R,14S,17R)-3,7-dihydroxy-10,13-dimethyl-12-oxohexadecahydro-1H-cyclopenta[a]phenanthren-17-yl)pentanoic acid	0.9749
2*	5Alpha-Androstan-3,17-Dione	0.9694
3	5’-Amino-5’-deoxyadenosine	0.8935
4	(2S,3S,4S,5R,6R)-6-[[(3S,4S,6aR,6bS,8aR,9R,12aS,14bR)-9-hydroxy-4-(hydroxymethyl)-4,6a,6b,8a,11,11,14b-heptamethyl-1,2,3,4a,5,6,7,8,9,10,12,12a,14,14a-tetradecahydropicen-3-yl]oxy]-5-[(2S,3R,4S,5R,6R)-4,5-dihydroxy-6-(hydroxymethyl)-3-[(2S,3R,4R,5R,6S)-3,4,5-trihydroxy-6-methyloxan-2-yl]oxyoxan-2-yl]oxy-3,4-dihydroxyoxane-2-carboxylic acid	-0.8623
5	7-Hexadecynoic acid	-0.8629
6	4-Ethyl-7,11-dimethyldodeca-trans-2-trans-6-1-o-trien-1-al	-0.8666
7	(1R,2R,5S,8R,14R,15R,16S)-16-hydroxy-1,2,14,17,17-pentamethyl-8-(prop-1-en-2-yl)pentacyclo[11.7.0.0]icosane-5,15-dicarboxylic acid	-0.8688
8	NCGC00385267-01_C15H20O4_2-(6-Hydroxy-3,8-dimethyl-2-oxo-1,2,4,5,6,7,8,8a-octahydro-5-azulenyl)acrylic acid	-0.8706
9	NCGC00169801-02_C30H46O4_Lanosta-8,24-dien-26-oic acid, 21-hydroxy-3-oxo-, (5xi,13alpha,14beta,17alpha,20S,24E)	-0.8710
10	13-HDoHE	-0.8740
11	His-Ile	-0.8746
12	Lys-Ile	-0.8802
13	3-acetoxy-4-(dimethylamino)butyric acid	-0.8831
14	(R)-4-((3R,5R,7R,8R,9S,10S,12S,13R,14S,17R)-7,12-dihydroxy-10,13-dimethyl-3-(sulfooxy)hexadecahydro-1H-cyclopenta[a]phenanthren-17-yl)pentanoic acid	-0.8834
15	Methyl trans-styryl ketone	-0.8896
16	22-Hydoxy-2-hopen-1-one	-0.8907
17	20-Hydroxy-6Z,15Z-eicosadienoic acid	-0.899
18	9(10)-EpOME	-0.9022
19	(4E,7E,10E,13E)-Hexadeca-4,7,10,13-tetraenoic acid	-0.9096
20	2-Butanone, 4-(2,6,6-trimethyl-2-cyclohexen-1-yl)	-0.9096
21	NCGC00381425-01!8-hydroxy-8-(3-octyloxiran-2-yl)octanoic acid [IIN-based on: CCMSLIB00000846585	-0.9138
22	9-OxoOTrE	-0.9205
23	Loliolide	-0.927
14	alpha-Pinene oxide	-0.9510
25*	((4R)-4-((3R,5S,7R,9S,10S,12S,13R,14S,17R)-3,7,12-trihydroxy-10,13 dimethylhexadecahydro-1H-cyclopenta[a]phenanthren-17-yl)pentanoyl)valine	-0.9879

The full names of the top 25 compounds identified by Pearson r pattern correlation with their corresponding correlation coefficients. Significance determined by ANOVA with Tukey’s *post-hoc* testing. *p < 0.05.

To assess associations between metabolite and microbiota changes, we performed 16S rRNA amplification and sequencing using DNA isolated from cecum samples collected on D3 after MI. *Bacteroidaceae*, *Lactobacillaceae*, *Clostridiaceae*, and *Eggerthellaceae* metabolize lithocholic acid and conjugate amino acids such as glycine and lysine, among others, to cholic acid (Lucas et al., 2021). When relative abundances were examined, a greater abundance of *Lactobacillaceae* was found in cecum samples from females when compared to males, **Figure 5D**. Ultimately, we reveal sex-specific differences in the abundance of secondary bile acid metabolites in the cecum on D3 after MI.

To further investigate microbiota-metabolite associations, we performed distance correlation analysis between family-level microbial relative abundances and annotated cecum metabolites (626 compounds) in MI D3 and NoSx samples (n=7 per group, both sexes pooled) to capture linear and non-linear associations (Székely et al., 2007). This analysis identified 950 significant microbe-metabolite associations (dCor > 0.5, *p* < 0.05) involving 19 bacterial families, **Figure 5E**. Hierarchical clustering of the distance correlation matrix resolved three vertically stacked blocks of bacterial families. A set of taxa, *Defluviitaleaceae, Anaerofustaceae, UCG_010, Christensenellaceae, Ruminococcaceae, Oscillospiraceae*, and *Tannerellaceae*, showed concordant positive associations with a shared metabolite panel. A second set of taxa of *Butyricicoccaceae, Bacteroidaceae, Lachnospiraceae, Lactobacillaceae, Prevotellaceae*, and *Marinifilaceae*, displayed a reciprocal, anti-correlated pattern against the same metabolites. This reciprocal structure supports the idea that the metabolite landscape shifts with groups of taxa changes. The metabolite signature shared by the two blocks is dominated by products of microbial aromatic amino acid catabolism, namely phenethylamine, tyramine, 3,4-dihydroxyphenylacetate (DOPAC), 4-vinylphenol, cinnamic acid, and shikimate (dCor = 0.55–0.79), together with the tryptophan catabolite indole-3-lactic acid (dCor = 0.63–0.71) and the amino-acid-containing peptides Glu-Val-Phe and Val-Trp (dCor = 0.49–0.77). The concordance between changes in the relative abundances of *Lactobacillaceae, Bacteroidaceae, Lachnospiraceae, Prevotellaceae*, and *Marinifilaceae* and of select metabolites on D3 after MI suggest that this group of taxa might be important in the gut microbiota-metabolome response to MI.

Lastly, pathway enrichment analysis, performed within the MMP module against the predicted metabolic capacity of the detected microbial taxa, identified several significantly enriched KEGG pathways (Gamma-adjusted *p* < 0.05), with a clear convergence on amino acid metabolism, **Table 4**. Free L-glutamate, L-glutamine, and L-asparagine were recurrent hits across alanine, aspartate and glutamate metabolism (*p* = 0.004), glyoxylate and dicarboxylate metabolism (*p* = 0.007), and cyanoamino acid metabolism (*p* = 0.020); lysine metabolism was enriched in parallel through lysine degradation (*p* = 0.002) and lysine biosynthesis (*p* = 0.052); and D-amino acid metabolism (*p* = 0.052) drew on both L- and D-glutamate and L-lysine. This peak-level amino-acid signal mirrors the compound-level heatmap pattern: the same MI-responsive families that correlated with tyramine, DOPAC, phenethylamine, indole-3-lactic acid, and the dipeptides Glu-Val-Phe and Val-Trp are families with well-characterized capacity for decarboxylation, deamination, and peptide turnover of these amino acid substrates. Additional significantly enriched pathways included propanoate metabolism (*p*-adjust = 0.002), purine metabolism (*p* = 0.001), pyrimidine metabolism (*p* = 0.001), amino sugar and nucleotide sugar metabolism (*p* = 0.004), and galactose metabolism (*p* = 0.020). Together these integrative analyses demonstrate that the post-MI microbial community restructuring is statistically linked to coordinated metabolite changes.

**Table 4:** Pathway enrichment analysis of cecal metabolites.

Pathway	Total	Hits	Sig. hits	Gamma-adjusted p-value	Sig. compounds
Purine metabolism	46	4	2	0.001	C00242; C05512
Pyrimidine metabolism	31	2	2	0.001	C00064; C00106
Lysine degradation	4	1	1	0.002	C00489
Propanoate metabolism	7	1	1	0.002	C05984
Alanine, aspartate and glutamate metabolism	13	6	3	0.003	C00152; C00025; C00064
Amino sugar and nucleotide sugar metabolism	24	6	2	0.004	C00329; C00645
Glyoxylate and dicarboxylate metabolism	13	4	2	0.007	C00025; C00064
Galactose metabolism	30	15	2	0.02	C05404; C00492
Cyanoamino acid metabolism	4	3	1	0.02	C00152
Lysine biosynthesis	13	7	2	0.05	C03972; C00047
D-Amino acid metabolism	12	10	3	0.05	C00025; C00217; C00047

Pathway enrichment was performed against the predicted metabolic capacity of the detected microbial taxa using the MMP module of MicrobiomeAnalyst 2.0 with the KEGG pathway database (Mus musculus). Total = number of compounds in pathway; Hits = detected compounds matching the pathway; Sig. Hits = significantly altered compounds; P-adjust = Gamma-adjusted *p*-value. Pathways with *p*-adjust < 0.05 are shown, along with D-Amino acid metabolism (*p*-adjust = 0.052) due to its biological relevance.

### 3.7 Sex-dependent changes in intestinal structure after MI

**Figure 6:**
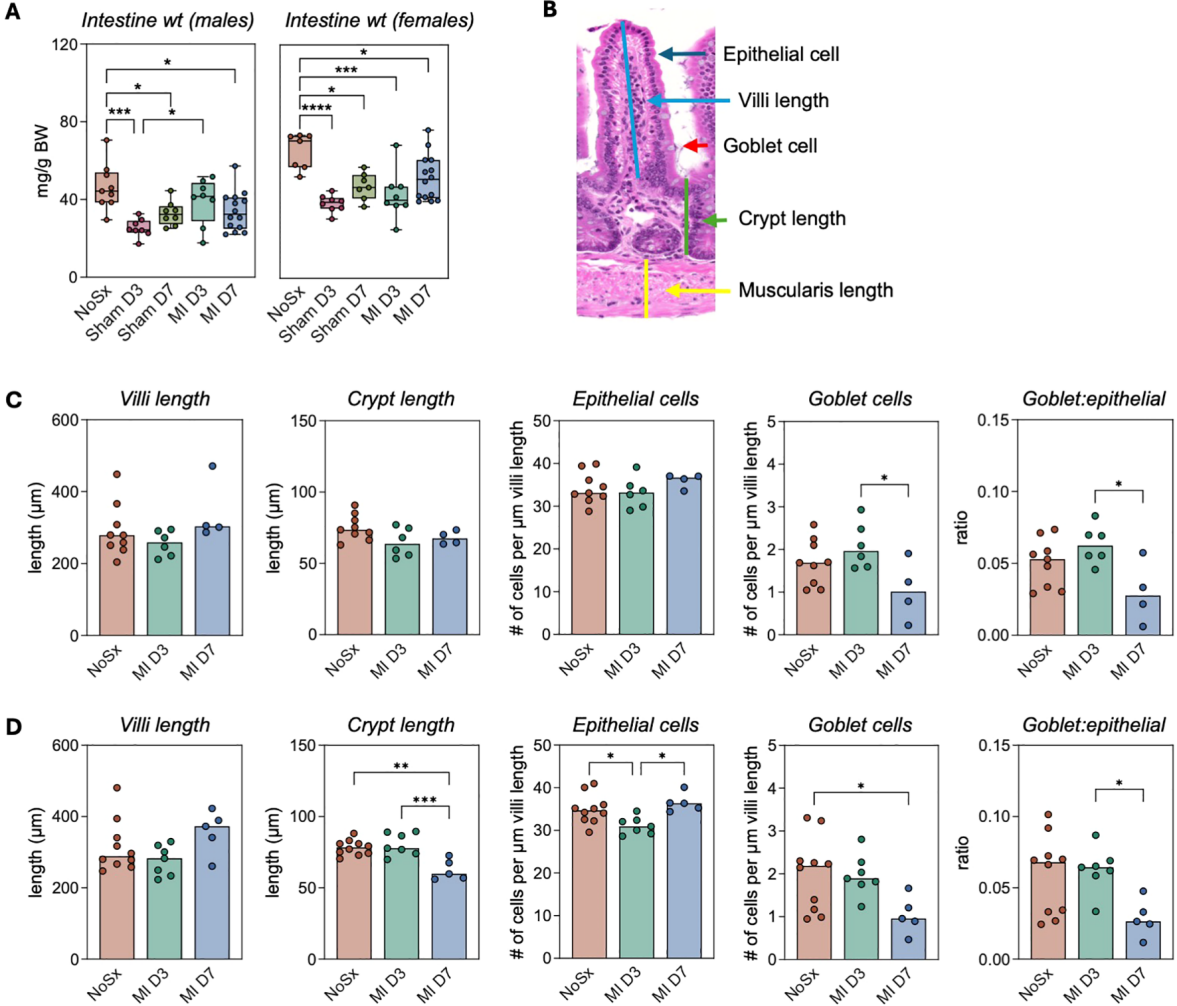
Changes to small intestine structure over time after MI in male and female mice. The small intestine was isolated, the contents extruded, external fat and Peyer’s Patches removed, and tissue weighed. Portions of the jejunum were fixed, embedded in paraffin, sectioned, stained with H&E, and digitally scanned. Scanned sections from NoSx, MI D3, and MI D7 cohorts were analyzed using QuPath v6.0. Each dot indicates the average result from the analyses of 3–8 villi from an individual mouse. **(A)** Whole small intestine weights indexed to body weight in males and females. Significance calculated using ANOVA and Tukey’s *post-hoc* test. *p < 0.05, ***p < 0.001, ****p < 0.0001. **(B)** A representative small intestine villus demonstrating the measurement of villi height, crypt thickness, muscularis thickness and identification of endothelial and goblet cells. **(C, D)** Differences in villi height, crypt length, epithelial and goblet cell numbers/µm villi, and the ratio of goblet:epithelial cell numbers in male **(C)** and female **(D)** mice at different timepoints after MI. Significance was calculated using ANOVA with Tukey’s *post-hoc* test. *p < 0.05, **p < 0.01, ***p < 0.001.

Given the observed differences in gut microbial populations and the established impact of gut microbiota on intestinal morphology and function, we examined small intestine weight and structure after euthanasia. We first measured the weight of the dissected small intestine in male and female mice from NoSx mice, indexed to body weight. Interestingly, baseline small intestine weight was sex-specific and lower in males than females, **Figure 6A**. After surgery, indexed small intestine weights in females were decreased after SH and MI surgeries when compared with NoSx cohorts. Male mice also showed decreased indexed small intestine weight after SH surgery, however, MI surgery only significantly impacted small intestinal weight on D7.

Since MI provoked the most profound changes to gut microbiota composition, we examined small intestine morphology following MI in greater detail. H&E-stained histological sections of the jejunum (collected from NoSx mice and MI mice euthanized on D3 and D7) were prepared, and structural components and epithelial and goblet cells, **Figure 6B**, were analyzed and quantified. In males, villi length and crypt thickness were not significantly altered after MI surgery, **Figure 6C**. Similarly, there was no change detected in villi length in females either, **Figure 6D**. However, females demonstrated reductions in intestinal crypt length on D3 and D7, indicative of some structural impact of MI, and a transient reduction in epithelial cell numbers on D3. In both males and females, the goblet:epithelial cell ratio was reduced on MI D7 when compared to NoSx samples, **Figures 6C, D**.

Collectively, these findings show that surgery results in decreased small intestine weight early after SH and MI surgery, with some morphological and cellular differences evident after MI. Moreover, we observed sex-specific differences: females exhibited greater alterations to crypt length and epithelial and goblet cells numbers after MI than males.

### 3.8 Transient intestinal immune cell expansions after MI, peaking on D3

**Figure 7:**
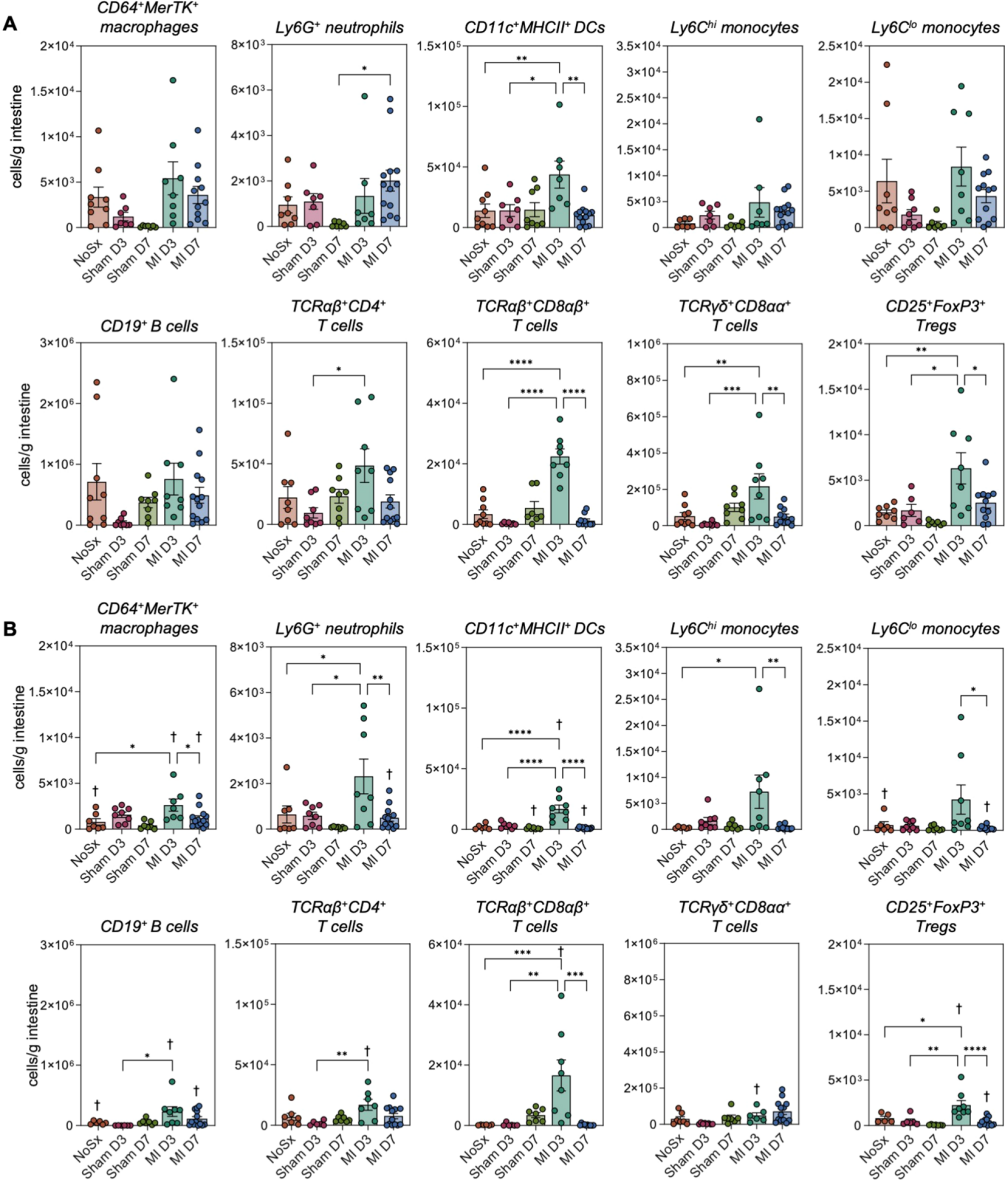
Innate and T cell populations in the small intestine. The small intestine was isolated, the contents extruded, external fat and Peyer’s Patches removed, and tissue weighed. A portion of the jejunum was weighed and used to prepare single cell preparations that were incubated with fluorescently labeled antibodies for analysis by flow cytometry. Flow data were analyzed using FlowJo software v10.10. Each dot indicates a sample from an individual mouse. These populations were identified and enumerated using the gating strategy shown in **Supplementary Figure 4**. Cell numbers normalized to total small intestine weight and are shown as numbers per gram of small intestine. **(A, B)** Differences in absolute numbers of innate cells (first row) and T cells (second row) cells per gram of intestine in male **(A)** and female **(B)** mice between NoSx and SH or MI at different timepoints. Y-axis scales are matched to allow for easier comparisons of cell numbers between males and females. Significance was calculated using ANOVA with Tukey’s *post-hoc* test. *p < 0.05, **p < 0.01, ***p < 0.001, ****p < 0.0001. Significant sex differences are indicated by a dagger symbol in **(B)**.

Given evidence that MI drives systemic inflammation and disrupts gut homeostasis (Lewis and Taylor, 2020; Rivera et al., 2024), we examined small intestinal immune cell populations after MI and SH in comparison to healthy, NoSx controls. Single cells from the intraepithelial compartment and lamina propria (LP) of the jejunum were isolated and absolute cell numbers quantified using multicolor flow cytometry, in male, **Figure 7A**, and female, **Figure 7B**, mice. Cell populations were identified and enumerated using the gating strategy shown in **Supplementary Figure 4**, with absolute cell numbers normalized to whole small intestine weight. Specifically, we enumerated myeloid populations in the intestinal LP, including CD64^+^MerTK^+^ macrophages, Ly6G^+^ neutrophils, CD11c^+^MHCII^+^ dendritic cells (DCs), and Ly6C^hi^ and Ly6C^lo^ monocytes, as well as lymphocytes such as CD19^+^ B cells, TCRαβ^+^CD4^+^ T cells, TCRαβ^+^CD8αβ^+^ T cells, and CD25^+^FoxP3^+^ regulatory T cells (Tregs) from the LP, alongside TCRγδ^+^CD8αα^+^ T cells, a population of intraepithelial lymphocytes (IELs) residing near the gut epithelium in the intraepithelial compartment.

Ultimately, no changes in immune cell numbers were detected after SH in males or females when compared to their NoSx. In contrast, both males and females showed significant expansions of CD11c^+^MHCII^+^ DC, TCRαβ^+^CD4^+^ T cell, TCRαβ^+^CD8αβ^+^ T cell, and CD25^+^FoxP3^+^ Treg populations after MI on D3, returning to levels comparable to NoSx controls by D7. Unique to MI males was a significant increase in TCRγδ^+^ CD8αα^+^ T cells on D3, while no variation in these cells was detected at any timepoint in females. Conversely, MI females exhibited selective increases in CD64^+^MerTK^+^ macrophage, Ly6G^+^ neutrophil, Ly6C^hi^ and Ly6C^lo^ monocyte, and CD19^+^ B cell populations on D3, with similar resolution to baseline, NoSx levels by D7. Additionally, female intestinal immune compartments displayed lower overall indexed cell numbers than males for most immune cell types at all time points, consistent across MI, SH, and NoSx groups, except for Ly6G^+^ neutrophils and Ly6C^+^ monocytes.

These data suggest that MI, but not SH, results in changes to immune cell abundances in the small intestinal compartment that are most pronounced D3 post-MI, with a return to control, NoSx levels by D7. Moreover, these dynamics are sex-specific, with females exhibiting generally lower intestinal immune cell numbers than males and distinct patterns of post-MI cell expansion across both myeloid and lymphocyte subsets.

### 3.9 Cardiac immune cell infiltration after MI but not SH

Successful wound healing after MI depends on the rapid response of resident innate cells and timely recruitment of additional immune cells from circulation into the injured myocardium (Crane et al., 2014; Latet et al., 2015; MacLeod and Mansbridge, 2016). To characterize the immune cell environments in the injured heart after MI, single-cell suspensions were prepared from whole heart tissue of NoSx, SH, and MI mice. Immune cells were characterized and quantified using multicolor flow cytometry, **Supplementary Figure 4**.

**Figure 8:**
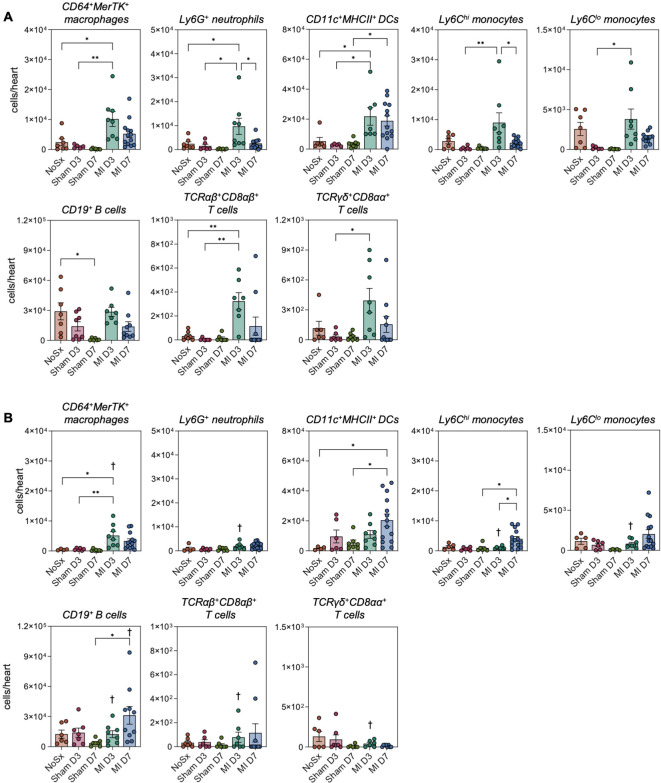
Innate and T cell populations in the heart. The heart was isolated, blood removed, and tissue weighed. Single cells suspensions were prepared with enzymatic tissue digestion and filtration and subjected to incubation with fluorescently labeled antibodies for flow cytometry analysis. Data were analyzed using FlowJo software v10.10. Each dot indicates a sample from an individual mouse. These populations were identified and enumerated using the gating strategy shown in **Supplementary Figure 4**. Cell numbers are shown as numbers per whole heart. **(A, B)** Differences in absolute numbers of innate cells (first row) and T cells (second row) cells per whole heart in male **(A)** and female **(B)** mice between NoSx and SH or MI at different timepoints. Y-axis scales are matched to allow for easier comparisons of cell numbers between males and females. Significance was calculated using ANOVA with Tukey’s *post-hoc* test. *p < 0.05, **p < 0.01. Significant sex differences are indicated by a dagger symbol in **(B)**.

No differences in immune cell numbers, except for CD19^+^ B cells in males, were found after SH in comparison to NoSx, **Figures 8A, B**. In contrast, MI males and females exhibited transient increases in CD64^+^MerTK^+^ macrophage cell numbers on D3, **Figure 8A, B**. Moreover, significant expansions in TCRγδ^+^CD8αα^+^ T cell, TCRαβ^+^CD8^+^ T cell, Ly6C^hi^ monocyte, CD11c^+^MHCII^+^ DC, and Ly6G^+^ neutrophil populations were recorded on D3 after MI in males, with cell numbers resembling baseline levels by D7, except for DCs. In contrast, no significant increases in any of these cell types were found in MI females on D3. Instead, we found delayed expansions of CD11c^+^MHCII^+^ DCs, Ly6C^hi^ monocytes, and CD19^+^ B cells on D7 after MI. Overall, our results show that immunological changes in the heart after MI surgery are sex-specific, with males showing significant, early expansions of innate cells and T cell subsets that are not observed by D7, while females exhibit delayed, reduced, and selective expansions of innate cells only.

### 3.10 Time-dependent changes in bone marrow immune compartment after MI

The bone marrow and spleen serve as key reservoirs for leukocyte production and mobilization after MI (Swirski and Nahrendorf, 2013). To investigate the dynamics of these immune cell compartments after SH and MI, single cell suspensions were prepared from femoral bone marrow and spleen tissue for characterization and quantification of immune cells using multicolor flow cytometry.

In male bone marrow, similar patterns of immune cell depletion on D3 and expansion on D7 were shared between SH and MI mice when compared to NoSx, namely in Ly6G^+^ neutrophil, CD64^+^MerTK^+^ macrophage, CD11c^+^MHCII^+^ DC, TCRαβ^+^CD4^+^T cell, TCRαβ^+^CD8αβ^+^T cell, and TCRγδ^+^CD8αα^+^ T cell populations, **Supplementary Figure 5A**. In females, these patterns were significant after MI on D7, **Supplementary Figure 5B**, and were often more pronounced than those found in males.

Spleens collected at euthanasia (D3) were weighed and indexed to body weight. At baseline, females had significantly greater indexed spleen weights than males across all groups, **Supplementary Figures 6A, C**. Male and female spleens showed significantly fewer immune cells in SH D3 mice when compared to all other cohorts, and increased numbers of CD64^+^MerTK^+^ macrophages and Ly6G^+^ neutrophils after MI, particularly on D7, **Supplementary Figures 6B, D**. Additionally, females showed lower overall numbers of Ly6G^+^ neutrophils, TCRαβ^+^CD4^+^ T cells, and TCRγδ^+^CD8αα^+^ T cells and greater numbers of CD64^+^MerTK^+^ macrophages and Ly6C^lo^ monocytes than their male counterparts, **Supplementary Figures 6B, D**. However, in both males and females, immune cell numbers in the spleen were largely unchanged after MI when compared to NoSx controls.

## 4 Discussion

The gut microbiome communicates with peripheral organ systems and modulates host physiological processes, including the immune response (Tang et al., 2017; Carrillo-Salinas et al., 2020). This study examines the gut-heart axis, a bidirectional communication between the intestinal microbiome and the cardiovascular system, in the context of cardiovascular injury, more specifically, an MI (Yang et al., 2015; Lam et al., 2016; Brown and Hazen, 2018; Carrillo-Salinas et al., 2020; Gutiérrez-Calabrés et al., 2020; Nesci et al., 2023; Zhao et al., 2023a). Expanding upon previous studies that have relied primarily on young, male animals, we used aging male and female mice to 1) characterize sex-specific differences in the systemic response to MI and 2) better represent the older population at higher risk for cardiovascular disease and adverse cardiac outcomes (Lindsey et al., 2021; Lucà et al., 2024; Pedretti et al., 2025). Here, we investigated the gut microbiome alongside physiological and immunological responses of the heart, small intestine, spleen, and bone marrow after a surgically induced MI in retired breeder C57BL/6N male and female mice, using SH surgery and NoSx as controls. Our findings indicate that changes in the gut microbiome, gut metabolome, and multi-organ immune responses are synchronized after MI surgery and are highly sex-specific.

### 4.1 MI triggers coordinated multi-organ responses synchronized with gut microbial dynamics

Permanent left anterior descending coronary artery ligation is a standard, reliable and reproducible murine model of nonreperfused MI, combining cardiac ischemic injury and systemic inflammation. This technique is well suited to investigate immunological interactions between organ systems and sex-differences in recovery from cardiac trauma (De Villiers and Riley, 2020; Lindsey et al., 2021). Inclusion of the SH surgery cohort enabled the distinction of responses attributable to surgical interventions alone, such as anesthesia, intubation, thoracotomy, and post-operativeanalgesia, from those driven by MI.

Wound repair and tissue regeneration after MI follows a conserved program (Brancato and Albina, 2011; Yan et al., 2013; Crane et al., 2014; Latet et al., 2015; MacLeod and Mansbridge, 2016; MacLeod and Mansbridge, 2016) that requires tight regulation of the timing and magnitude of immune cell infiltration. An overactive immune response can exacerbate injury and promote adverse cardiac remodeling, while an ineffective response prevents successful repair of damaged tissues (Frangogiannis, 2012; Gao et al., 2012; Myerburg and Junttila, 2012; Yan et al., 2013). Resident and infiltrating leukocytes clear necrotic tissue and debris, shape the inflammatory milieu, and orchestrate the repair and regeneration of damaged tissue, determining infarct size and extent of LV remodeling (Frangogiannis, 2012). Within hours of artery ligation, downstream cardiomyocytes and other cardiac cells perish, releasing damage associated molecular patterns (DAMPS) that activate an early inflammatory response beginning with neutrophil migration to the site of injury (Zhou et al., 2024). In young, male animal models of MI with prolonged ischemia, neutrophils dominating the early infarcted myocardium peak around day 1–3 after MI, accompanied by a massive infiltration of pro-inflammatory (Ly6C^hi^) monocytes/macrophages, and later, DCs (Dutta and Nahrendorf, 2015; Lai et al., 2019; Rusinkevich et al., 2019; Kologrivova et al., 2021; Simões and Riley, 2022). Macrophage infiltration peaks between days 3-5, while DC accumulation often reaches a peak between days 5-7 (Yan et al., 2013; Rusinkevich et al., 2019). Our findings in aging male and female mice largely follow this established temporal pattern, showing anticipated increases in neutrophils, macrophages, Ly6C^hi^ monocytes, and DCs, peaking on D3 after MI, with DC numbers stable or increased on D7, that were absent in SH controls. These data indicate that advanced age does not markedly impact the temporality of early innate immune cell recruitment to the infarct in this model.

The bone marrow and spleen generate and serve as major reservoirs for immune cells that are mobilized and recruited by the injured myocardium after MI to participate in inflammation and wound repair (Swirski et al., 2009; Dutta and Nahrendorf, 2015). While no coordinated changes to immune cell populations were found in the spleen, transient depletion of innate and adaptive cells in the bone marrow is consistent with bone marrow-driven emergency hematopoiesis and mobilization of leukocytes as the primary source of infiltrating cells after MI (Zhang et al., 2023). In our aging mouse model, the spleen appears to play a less prominent role than reported in some younger MI models.

As mentioned previously, permanent LAD ligation induces LV dilation and functional deficits after surgery (Cavasin et al., 2004; Pullen et al., 2020; Haider et al., 2025). In this study, MI mice exhibited significantly greater LV volume/area and decreased fractional area change (FAC) compared to controls on D3, although changes to LV volume/area were delayed to D7 in MI females. FAC is a quantitative measure of the percentage of LV wall that is contracting during systole, and its reduction is a marker of LV systolic dysfunction (Castiglioni et al., 2015). Similarly, greater LV volume and area are a direct measure of LV dilation after MI and generally indicate early adverse LV remodeling, secondary to a substantial loss of contractile myocardium and wall thinning in the infarct zone (Sutton and Sharpe, 2000; Frantz et al., 2022). These measurements substantiate the success of the MI-inducing surgery in our protocol and confirm preserved cardiac structure/function in SH mice. Furthermore, no changes in other standard measures of cardiac function, such as cardiac output, stroke volume, and aortic velocity-time integral (Ao VTI), were observed in the acute period after MI, indicating that noninfarcted areas of the heart compensated sufficiently to maintain normal blood flow. This preserved LV systolic function suggests that it is unlikely that the observed changes in intestinal microbiome, morphology, and immune cell populations are downstream results of intestinal hypoxia or under-perfusion at this early juncture. However, this compensation is temporary. Over the following few weeks, the remaining viable heart muscle will decompensate and will be unable to maintain ventricular systolic function, leading to overt heart failure and organ hypoperfusion (Lindsey et al., 2021).

While both MI and SH surgeries resulted in significant gut microbiome restructuring, MI produced more pronounced and coordinated alterations, suggesting that myocardial injury was the principal driver of disrupted gut microbial homeostasis. Moreover, despite modest, transient alterations to gut microbiota, immune cell populations in the heart, intestine, bone marrow, and spleen were comparable between NoSx and SH groups, indicating an absence of overt immune activation after SH surgery. Our protocol involved short-term exposure to anesthesia and a single administration of slow-release buprenorphine, however, more complex, isolated perioperative protocols, involving food withdrawal, prolonged anesthetic exposure (4 hours), Cefazolin prophylaxis, and repeated injections of analgesia, found significant reductions in gut microbial diversity and changes in bacterial taxonomic composition that were resolved within days of surgery (Serbanescu et al., 2019; Serbanescu et al., 2026). Combined, these data suggest that moderate perioperative interventions, without major tissue or organ damage, are sufficient to induce transient alterations in gut microbial community structure but not to incite a significant immune response.

Conversely, MI surgery produced distinct post-surgical gut microbial and metabolomic profiles, with many features shared between male and female cohorts. Specifically, MI was associated with significant expansions of *Bacteroidaceae* and *Tannerellaceae*, and an acute increase in *Marinifilaceae* populations on D3, likely a result of stress-induced opportunism. These findings indicate an MI-specific response in the gut microbiome that is independent of sex, and are consistent with reports of increased *Tannerellaceae* and *Marinifilaceae* in young male mice after MI (Guo et al., 2024), as well as associations between coronary artery disease and altered levels of *Bacteroidaceae*, *Marinifilaceae*, and *Tannerellaceae* in human patients (Nakajima et al., 2022). This expansion of Gram-negative *Bacteroidaceae, Marinifilaceae*, and *Tannerellaceae*, members of which have established proinflammatory potential and capacity for lipopolysaccharide (LPS)-driven immune activation, may indicate functional reprogramming of the gut ecosystem toward a potentially dysbiotic, inflammatory intestinal state.

The small intestine serves as a major reservoir of innate and adaptive immune cells that coordinate mucosal immunity and maintain the intestinal barrier (Ramanan et al., 2023; Meng et al., 2024; Mohamed et al., 2024). Antigenic and metabolic signals from resident intestinal microbiota can alter these immune compartments and shape the mucosal immune response (Yang and Cong, 2021; Muske and Knoop, 2023). Gram-negative bacteria-derived LPS, among other microbial antigens, are known, potent activators of mucosal DCs and macrophages, which subsequently promote the priming and expansion of CD4^+^ and CD8^+^ T cells through antigen presentation (Mohamadzadeh et al., 2005; Biscari et al., 2022; Wang et al., 2023). Indeed, we observed robust increases in DCs, macrophages, and CD8^+^ T cells in the small intestine on D3 after MI in both males and females, temporally aligned with the MI-associated expansion of Gram-negative *Bacteroidaceae, Marinifilaceae*, and *Tannerellaceae*. Moreover, intestinal *Marinifilaceae*expansion has been linked to increased inflammation and intestinal injury in DSS-induced colitis (Shi et al., 2024), and thus, may similarly be contributing to the inflammatory milieu of the small intestine detected on D3 after MI. A concomitant increase in intestinal Tregs was also recorded on D3, which likely reflects an attempt to restore mucosal homeostasis as a compensatory response to intestinal inflammation. Some members of the *Bacteroidaceae* family, such as *Bacteroides fragilis*, may also regulate intestinal inflammation by driving CD25^+^ and FoxP3^+^ Treg differentiation via capsular polysaccharide A and other metabolites, raising the possibility that *Bacteroidaceae* in the post-MI gut may simultaneously contribute to amplified proinflammatory DC-mediated T cell activation through LPS and engage immunoregulatory Treg pathways (Round and Mazmanian, 2010; Johnson et al., 2015). This MI-associated dysbiosis and concomitant intestinal inflammation persists throughout the first week of recovery, although partial reversions toward baseline microbiota composition and intestinal immune cell burden is evident by D7. These immune responses were absent in the less dysbiotic, control SH surgery group.

By D7, significant goblet cell depletion was present in the small intestine of both males and females, suggesting damage to the intestinal barrier. Goblet cells are central to mucus production and mucosal immunity, and their depletion is associated with loss of barrier function and inflammation in many settings (López Cauce et al., 2020; Yang and Yu, 2021). We speculate that the early post-MI expansion of *Bacteroidaceae* and *Tannerellaceae*, which contain several known mucin-degrader taxa (Glover et al., 2022; Pan et al., 2022), may decompensate the mucus barrier, leading to goblet cell exhaustion. When intestinal barrier integrity is perturbed, Gram-negative populations, like *Bacteroidetes*, are favored, which supply large amounts of LPS that engage toll-like receptors (TLRs) on macrophages and DCs (Wang et al., 2023). The temporal concordance between MI-associated intestinal dysbiosis, mucosal dysfunction, and immune cell expansion, combined with the absence of significant intestinal immune response after SH surgery, suggests that gut immunity is mobilized as part of the inflammatory response to MI, rather than a result of generic postoperative inflammation. This implies that specific MI-associated cues, perhaps including those produced by cardiomyocytes and other cells damaged by ischemia, are required to active the intestinal immune compartment, thereby supporting the existence of a directed gut-heart-immune axis in MI.

To identify relationships between microbial taxa (microbiota) and metabolites (metabolome) following MI, we performed distance correlation analyses between family-level taxonomic abundances and annotated cecal, pooling male and female samples to isolate non-sex specific, MI-driven effects. These relationships provide insight into how shifts in microbial composition are linked to changes in metabolic output in the small intestine, highlighting potential functional roles of the microbiome following MI. Ultimately, three structured taxa-metabolite groups were identified: homeostatic, MI-expanded and neutral. The homeostatic block groups seven families (*Defluviitaleaceae, Anaerofustaceae, UCG_010, Christensenellaceae, Ruminococcaceae, Oscillospiraceae*, and *Tannerellaceae*) which are depleted or stable after MI. In contrast, the MI-expanded block groups six MI-expanded families (*Butyricicoccaceae, Bacteroidaceae, Lachnospiraceae, Lactobacillaceae, Prevotellaceae, Marinifilaceae*). These two blocks, homeostatic and MI-expanded, share a common metabolite panel dominated by products of microbial aromatic amino acid catabolism, including phenethylamine, tyramine, DOPAC, 4-vinylphenol, cinnamic acid, and the tryptophan catabolite indole-3-lactic acid, as well as dipeptides containing glutamate, valine, phenylalanine, and tryptophan. Phenethylamine and tyramine are decarboxylation products of tryptophan and phenylalanine catalyzed by bacterial aromatic amino acid decarboxylases (Williams et al., 2014); indole-3-lactic acid is a *Lactobacillaceae*- and *Bifidobacterium*-derived tryptophan catabolite (Roager and Licht, 2018; Ehrlich et al., 2020); and the dipeptide signal reflects peptide-turnover activity of *Bacteroidaceae* and *Lactobacillaceae* (Lucas et al., 2021). The remaining block of taxa*, Deferribacteraceae, Peptococcaceae, Monoglobaceae, Desulfovibrionaceae, Akkermansiaceae*, is neutral and maybe inconsequential as it shows uniformly weak correlations, suggesting that their abundance changes are unlikely to be either driving or driven by the annotated metabolite pool. However, they may act through metabolites outside the current annotated 626-compound library.

A second metabolite panel, exclusive to the homeostatic block, and absent in MI-expanded block, is consistent with a coordinated reciprocal restructuring of the metabolome rather than independent drift. Members of the homeostatic block correlate specifically with anti-inflammatory host lipid mediators (FAHFA 5:0/20:0, DG 22:5n6/22:6n3, TAG 44:7, sphingomyelin) and microbial secondary metabolites (cyclo(Phe-Leu), phenylacetic acid, epsilon-decalactone, terpenoid-class compounds) which were entirely uncorrelated with the taxa in the MI-expanded block. FAHFAs suppress TLR4-driven inflammation through GPR120 (Yore et al., 2014), and their association with *Ruminococcaceae* and *Christensenellaceae* is consistent with butyrate-driven regulation of host lipid mediator synthesis. The diketopiperazine cyclo(Phe-Leu) and related microbial secondary metabolites have established roles in quorum sensing and modulation of host mucosal immunity (Holden et al., 1999). Because none of the MI-expanded taxa replace this output, the post-MI dysbiosis creates a FAHFA/secondary-metabolite deficit that is invisible if only the aromatic amino acid catabolism panel is examined, and that may contribute independently to the impaired mucosal barrier and immune dysregulation observed after MI.

Pathway enrichment against the predicted metabolic capacity of the detected taxa expanded upon the correlations and revealed a convergence on amino acid metabolism. Six of the significantly enriched pathways, alanine, aspartate and glutamate metabolism, glyoxylate and dicarboxylate metabolism, cyanoamino acid metabolism, lysine degradation, lysine biosynthesis, and D-amino acid metabolism, repeatedly showed L-glutamate, L-glutamine, L-asparagine, and L-lysine as significant hits. These free amino acid substrates feed directly into the bacterial decarboxylation, deamination, and peptide-turnover reactions whose products (tyramine, phenethylamine, DOPAC, indole-3-lactic acid, Glu-Val-Phe, Val-Trp) define the homeostatic/MI-expanded shared metabolite panel in the heatmap, linking the peak-level pathway signal to compound-level evidence of altered microbial amino acid handling. The same families whose peptide correlations define this signature, *Bacteroidaceae* and *Lactobacillaceae*, are those with well-characterized amino acid conjugation of bile acids (Lucas et al., 2021), the biochemistry underlying the sex-specific detection of cholylvaline in males after MI. In parallel, enrichment of propanoate metabolism (*p*-adjust = 0.002) points to altered short-chain fatty acid output by the expanded *Lachnospiraceae* and *Bacteroidaceae* (Reichardt et al., 2014; Louis and Flint, 2017). Although individual SCFAs were below the detection limit of our untargeted platform, pathway enrichment provides indirect evidence of SCFA remodeling, which would in turn modulate the increase in mucosal Treg and macrophage numbers we observed (Arpaia et al., 2013; Smith et al., 2013). Enrichment of galactose metabolism (*p*-adjust = 0.020), together with amino sugar and nucleotide sugar metabolism (*p*-adjust = 0.004), is consistent with altered microbial degradation of host mucin glycans, aligning with the goblet cell depletion observed in histologically.

### 4.2 Biological sex shapes the response to MI

Prior to surgical intervention, male and female mice showed distinct gut microbial profiles despite a common vendor, shared housing conditions, and identical pre-operative treatment, consistent with reports of sexual dimorphism in the gut microbiome. At baseline, sex-specific configurations of intestinal *Lactobacillaceae*, *Muribaculaceae*, *Erysipelotrichaceae*, *Lachnospiraceae*, *Bacteroidaceae*, and *Oscillospiraceae* were recorded. Many of these bacterial families have been reported as sex- or hormone-sensitive in prior murine studies (Son et al., 2019; McGee and Huttenhower, 2021), though specific family-level patterns vary depending on study design. Additionally, the male jejunum housed larger populations of resident macrophages, B cells, and Ly6C^lo^ monocytes than females at baseline, indicating a higher steady−state myeloid and B−cell set point. Collectively, our findings demonstrate that sex influences multiple facets of the murine gut, consistent with observations in humans (Salazar et al., 2017).

In male hearts, MI drove robust but transient increases in multiple cardiac T cell subsets and innate populations on D3, indicating greater early cardiac inflammation. These responses corresponded to earlier, more pronounced LV dilation and functional decline than females, indicative of more rapid adverse remodeling. In contrast, females exhibited only a modest early elevation in cardiac macrophages, with delayed recruitment of DCs and Ly6C^hi^ monocytes, concurrent with delayed LV dilation. Overall, female hearts transitioned to adverse remodeling in a slower, more controlled manner, associated with a less exaggerated inflammatory immune response in the heart. The temporal alignment of immune cell infiltration and LV remodeling underscores the importance of both the magnitude and timing of leukocyte recruitment in shaping cardiac remodeling after MI.

Like in the heart, male cohorts also exhibited worse physiological recovery and more pronounced gut microbial responses to surgery than their female counterparts. Both MI and SH surgery elicited early decreases in intestinal, cecum, and body weight in both males and females, reflective of the physiological impacts of surgical intervention rather than cardiac dysfunction alone. In animal studies, dramatic weight loss after surgery may result from a decrease in appetite and nutritional status, leading to a loss of body condition (Chappell et al., 2011; Talbot et al., 2020), while changes to intestinal and cecum content weight may also reflect altered gut microbial composition and activity (Bolsega et al., 2023). Throughout, females showed better maintenance of weight over time, potentially indicative of improved nutrition and productive recovery responses after MI.

Alongside sustained reductions in body and organ weights, males experienced more significant gut microbiota alterations with a loss in microbial diversity after MI. Acute post-MI gut dysbiosis was strongly sex specific, such that males showed roughly 10-fold larger increases in *Marinifilaceae* populations than females and double the burden of *Bacteroidaceae* in the gut, alongside a characteristic increase of *Coprobacillaceae* and a marked loss of butyrate-producing *Lachnospiraceae*. An increased abundance of gram-negative *Bacteroidaceae* and *Marinifilaceae* has been associated with decreased microbiome diversity, intestinal and systemic inflammation, and even the onset of inflammatory disease (Forbes et al., 2016; Manor et al., 2020; Stojanov et al., 2020; Maciel-Fiuza et al., 2023; Shi et al., 2024). Similarly, a depletion of *Lachnospiraceae*, a family comprising major SCFA producers linked to anti-inflammatory activity, is frequently observed in intestinal dysbiosis and inflammatory diseases (Zhang et al., 2019; Ćesić et al., 2023), as well as in both human and mouse studies of heart failure and coronary disease (Amiri et al., 2021; Khannous-Lleiffe et al., 2021; de Wit et al., 2024). Specific to cardiac injury, *Lachnospiraceae* was consistently depleted in young male C57BL/6J mice after permanent MI and transverse aortic constriction (de Wit et al., 2024), and in older human patients after an ST-elevated MI (You et al., 2025), heart failure, and other cardiovascular diseases (Jie et al., 2017; Kummen et al., 2018; Zhu et al., 2018; de Wit et al., 2024). While *Bacteroidaceae* are diverse, the enrichment of *Marinifilaceae* with *Bacteroidaceae* alongside a loss of *Lachnospiraceae* may reflect a more pro-inflammatory, stress-associated dysbiosis associated with cardiovascular disease.

Conversely, the female gut was associated with a 10-fold larger increase in *Akkermansiaceae*, double the abundance of *Lactobacillaceae* as males, and characteristically higher *Desulfovibrionaceae*, *Prevotellaceae*, and *Eubacteriaceae* populations, with partial retention of *Lachnospiraceae*. However, while *Lactobacillaceae* remained relatively more abundant in females than males, females experienced an overall reduction in *Lactobacillaceae* populations after MI when compared to their baseline microbiota. Both *Akkermansiaceae* and *Lactobacillaceae* are promising probiotics linked to better status of health, with functions involved in the alleviation of inflammation and restoration of the intestinal barrier through the secretion of beneficial metabolites (Lobionda et al., 2019; Aoun et al., 2020; Segers and de Vos, 2023). Increased *Akkermansiaceae* abundance is associated with reduced vascular and systemic inflammation and protection against cardiac remodeling, and supplementation improves left ventricular function in ApoE-/- mice while reducing risk of atherosclerosis and ischemic heart disease (Gofron et al., 2024; Xiao et al., 2024). *Desulfovibrionaceae* are sulfate-reducing, LPS-producing pathobionts often associated with intestinal dysbiosis, barrier disruption, and mucosal immune activation (Collisson et al., 2014; Chang et al., 2015). Hydrogen sulfide, a metabolic byproduct of sulfate-reducing bacteria, is a main causative agent for morphological and functional damage to the gut epithelia in gastrointestinal disease (Huang et al., 2024). Consequently, a decrease in barrier-protective *Lactobacillaceae* combined with an overgrowth of mucin-degrading *Akkermansiaceae* and potentially pathogenic *Desulfovibrionaceae* may lead to epithelial damage and an overconsumption of mucin after MI, resulting in a thinner mucus layer and impaired barrier function. Indeed, MI appeared to induce greater intestinal remodeling in females than in males, as characterized by reduced villi crypt length, goblet cell depletion, and transient epithelial loss.

While male mice exhibited more aggressive cardiac inflammation, females mounted a stronger, contained mucosal innate immune response after MI. Marked expansions of intestinal macrophages, neutrophils, DCs, and Ly6C^hi^ monocytes were found in response to MI in females, whereas male intestines were only selectively enriched in DCs. An accumulation of innate effector cells can promote epithelial apoptosis and villus shortening through the release of proinflammatory mediators, such as TNF (Parker et al., 2019), providing a plausible mechanism for greater intestinal remodeling in females after MI. Both sexes mounted comparable adaptive responses to MI, although male cohorts showed a characteristic increase in TCRγδ^+^ T cells, suggesting that γδ T cell-dependent pathways were discriminately engaged in the male murine gut. Sex-specific hormonal and microbial environments in the intestine likely constrain γδ T cell expansion in females, consistent with known sex-differences in mucosal T cell activation (Elderman et al., 2018; Hoffmann et al., 2023), though these parameters have not yet been investigated in intestinal γδ T cells.

Consistent with changes to gut bacterial populations, metabolomics revealed sex-specific secondary bile acid profiles on D3 after MI. Males showed a differential abundance of intestinal cholylvaline, a microbially conjugated bile acid, while 12-oxo-lithocholic acid (12-oxo-LCA), a fecal secondary bile acid, was predominant in females. No differences in deoxycholic acid or chenodeoxycholic levels were found in males versus females, suggesting that the male-specific increase in cholylvaline and female-specific increase in 12-oxo-lithocholic acid were not secondary to sex-specific differences in their substrates. Re-conjugation of liver-derived chenodeoxycholic and cholic acids with amino acids such as taurine, glycine, and, to a lower extent, valine, as well as the modification of chenodeoxycholic acid into secondary bile acids like lithocholic acid are known functions of the host and gut microbiota (Wahlström et al., 2016; Lucas et al., 2021; Ay et al., 2022; Mohanty et al., 2024; Varanasi et al., 2025). Taxa with prominent amino acid conjugation activities include *Lachnospiraceae* and *Bacteroidaceae* (Lucas et al., 2021), and their higher relative abundance in male versus female microbiota correspond to greater cholylvaline in the male intestinal lumen. In contrast, increases in the secondary bile acid 12-oxo-LCA were associated with increased *Clostridiaceae*, poor pro-inflammatory NLRP3-inflammasome induction when compared with 11-oxo-LCA, and an anti-inflammatory effect during DSS-induced colitis through reduction of IL-17 from ILC3 cells (Alimov et al., 2019; Li et al., 2023; Luo et al., 2025). Studies of ulcerative colitis in human and mice found reduced 12-oxo-LCA correlated with increased disease and reduced *Clostridium* species (Yang et al., 2021; Li et al., 2023). Selectively increased 12-oxo-LCA in the female intestinal lumen after MI is coincident with the presence of reduced overall immune cell accumulation in multiple tissues and a greater abundance of intestinal *Clostridiaceae*. Together we speculate that these metabolites may play signaling roles in immune modulation, metabolism, and inflammation, potentially contributing to the observed sex-specific inflammatory patterns. To our knowledge, no prior studies have examined the effects of cholylvaline accumulation on immune cells or intestinal barrier integrity. Our findings here point to a potential role for this metabolite in shaping male responses to MI-related stress and injury.

### 4.3 Conclusions

Overall, our data show that MI, but not SH surgery, elicits coordinated immune responses across intestinal, cardiac, and hematopoietic compartments that are temporally aligned with gut microbiome remodeling. Our findings reflect interconnected, rather than independent and organ-specific, responses to MI. Notably, day 3 post-MI consistently appears as a critical timepoint of peak multi-organ activity, with responses largely resolving by day 7. Moreover, integrative microbiome-metabolome analyses demonstrate that post-MI microbial community restructuring is not merely compositional but is statistically linked to coordinated metabolite changes converging on pathways relevant to immune modulation, SCFA signaling, amino acid conjugation, and mucosal barrier function. These findings provide a mechanistic framework connecting the gut dysbiosis observed after MI to intestinal morphology and multi-organ immune compartment alterations described in this study.

Importantly, the response to MI is strongly modulated by sex: males exhibit greater early cardiac inflammation, accelerated adverse cardiac remodeling, and more pronounced gut dysbiosis, whereas females display more controlled and/or delayed immune responses, greater intestinal remodeling, and better maintenance of physiological homeostasis. Distinct, sexually dimorphic microbiome and metabolomic profiles are revealed, highlighting sex-specific microbiome-metabolome signatures that may differentially shape inflammatory and recovery trajectories.

### 4.4 Significance

Collectively, these data are consistent with growing evidence that cardiac injury, such as MI, can actively drive gut microbiome remodeling, while disrupted intestinal homeostasis aggravated by cardiac injury can influence myocardial inflammation and impact cardiac repair (Carrillo-Salinas et al., 2020; Zhao et al., 2023a; Chen et al., 2025). This creates a bidirectional axis of intestinal and cardiovascular dysfunction. Our findings further suggest that this gut-heart axis is fundamentally shaped by sex, with implications for individual variability in post-MI recovery and for the development of sex-informed therapeutic strategies. Our pre-clinical data may also be relevant to otherwise healthy older adults experiencing MI or other cardiovascular disease/injury, as our model is designed to more accurately reflect the aging human population.

Additionally, although we focus on MI, the surgical nature of our model may permit broader applicability to other surgical settings. Currently, the Enhanced Recovery After Surgery protocol encourages pre-operative carbohydrate loading as an element promoting good post-surgical outcomes after many surgeries, including reparative cardiac surgeries (Jiménez et al., 2011; Umezawa Makikado et al., 2013; Flordelís Lasierra et al., 2015; Arora et al., 2018; Noss et al., 2018; Coleman et al., 2019; Engelman et al., 2019; Ackerman et al., 2020; Zaouter et al., 2022). However, most carbohydrate loading solutions contain readily absorbed simple sugars which do not reach and, thus, do not support the gut microbiota, and contain minimal to no fiber. Our data supports the idea that improved pre-surgery or peri-operative dietary nutrition to enhance gut health may improve patient outcomes after an MI or after cardiac or other surgeries.

### 4.5 Limitations

The mice used here were healthy when the study began and consumed a nutritionally optimized diet. This is not representative of the clinical population, in which surgical patients often present with co-morbidities, associated with pre-existing gut dysbiosis, and malnourishment associated with a high fat, high calorie, and low fiber diet.

We did not compare recovery longer than 7 days after surgery and, thus, cannot comment on the long-term stability and impact of changes in the gut microbiome or immune microenvironments.

None of the mice demonstrated reductions in cardiac output or stroke volume indicating that organ perfusion was likely adequate at this early time. However, we did not perform pulsed wave Doppler analyses in arteries in the lower limbs to gain insight into blood flow into the intestine or secondary immune organs, like the spleen or bone marrow.

We recognize that short chain fatty acids may have contributed to the observed changes in immune cell changes detected. However, these small metabolites were not detected in our metabolome data set so we cannot comment on their involvement in immune regulation.

Finally, although we have identified bacterial taxa which were significantly different in MI versus SH, we have not tested whether supplementation of bacteria reduced in MI would improve any outcomes in recipient mice.

## Data Availability

The raw data supporting the conclusions of this article will be made available by the authors, without undue reservation.

## Supplementary material

Supplementary Figure 1 Impact of surgery on total body and cecum weight. Mice were weighed before surgery and on day 3 (D3) or day 7 (D7) after surgery. At euthanasia, the cecum was dissected and contents weighed. Each dot indicates a sample from an individual mouse. **(A)**. Actual body weights of male and female mice before surgery. Significance testing used an unpaired Student’s t-test. ****p <* 0.001. **(B)** Percent body weight change (relative to NoSx controls) was in SH and MI mice at different timepoints after surgery. **(C)** Cecum weight indexed to body weight are shown. For B and C, significance was calculated using ANOVA with Tukey’s *post-hoc* test. **p <* 0.05, ***p <* 0.01, ****p <* 0.001, *****p <* 0.0001.

Supplementary Figure 2 Changes in gut microbiota at several different timepoints after MI in males. Fecal pellets were collected from male mice on the day of surgery (D0) and the indicated days after surgery. 16S rRNA sequencing of fecal DNA enabled microbiota profiling. Analyses were performed using MicrobiomeAnalyst v2.0. Each dot indicates a sample from an individual mouse. **(A)** Differences in alpha (within-sample) diversity after MI at different timepoints. Chao1 and Shannon indices of alpha diversity reflect taxonomic richness (Chao1 and Shannon) and evenness (Shannon) of microbiota. Significance was calculated using one-way ANOVA and Tukey’s *post-hoc* test. ***p <* 0.01. **(B)** Bray-Curtis Index with PERMANOVA was used to calculate beta (between-sample) diversity in microbiota samples collected at different timepoints after MI. Results are visualized by spatial clustering on PCoA plots. **(C)** A heatmap shows bacterial family relative abundance. Stars indicate members of the *Oscillobacteraceae* family. **(D)** Relative abundance of the most discriminative bacterial families in MI samples. Significance was calculated using ANOVA with Tukey’s *post-hoc* test. **p <* 0.05, ***p <* 0.01, ****p <* 0.001, *****p <* 0.0001.

Supplementary Figure 3 Changes in gut microbiota at several different timepoints after MI in females. Fecal pellets were collected from female mice on the day of surgery (D0) and the indicated days after surgery. 16S rRNA sequencing of fecal DNA enabled microbiota profiling. Analyses were performed using MicrobiomeAnalyst v2.0. Each dot indicates a sample from an individual mouse. **(A)** Differences in alpha (within-sample) diversity after MI at different timepoints. Chao1 and Shannon indices of alpha diversity reflect taxonomic richness (Chao1 and Shannon) and evenness (Shannon) of microbiota. Significance was calculated using one-way ANOVA and Tukey’s *post-hoc* test (*p <* 0.05). **(B)** Bray-Curtis Index with PERMANOVA was used to calculate beta (between-sample) diversity in microbiota samples collected at different timepoints after MI. Results are visualized by spatial clustering on PCoA plots. **(C)** A heatmap shows bacterial family relative abundance. Stars indicate members of the *Oscillobacteraceae* family. **(D)** Relative abundance of the most discriminative bacterial families in MI samples. Significance was calculated using ANOVA with Tukey’s *post-hoc* test. **p <* 0.05, ***p <* 0.01, ****p <* 0.001, *****p <* 0.0001.

Supplementary Figure 4 Flow cytometry gating strategies. Flow cytometry was performed using the BD LSRFortessa Cell Analyzer and associated DIVA software. Data were analyzed using FlowJo software v10.10. The gating strategy to identify innate and T cell types in all tissues used. **(A)** Common gating strategy to isolate live, CD45^+^, single cell, immune cells in all panels. **(B)** Gating strategy to identify innate macrophage, monocyte, DC, and neutrophil immune cells. **(C)** Gating strategy to identify B cells, CD4^+^ and CD8^+^ T-cell subtypes. **(D)** Gating strategy to identify CD25^+^FoxP3^+^ Tregs.

Supplementary Figure 5 Innate and T cell populations in the bone marrow. Bone marrow from the left femur was flushed and cells collected, filtered, and subjected to incubation with fluorescently labeled antibodies for flow cytometry. Flow cytometry was performed using the BD LSRFortessa Cell Analyzer and associated DIVA software. Data were analyzed using FlowJo software v10.10. Each dot indicates a sample from an individual mouse. These populations were identified and enumerated using the gating strategy shown in **Supplementary Figure 4**. **(A, B)** Differences in absolute numbers of innate cells (first row) and T cells (second row) cells per femur in male **(A)** and female **(B)** mice between NoSx and SH or MI at different timepoints. Y-axis scales are matched to allow for easier comparisons of cell numbers between males and females. Significance was calculated using ANOVA with Tukey’s *post-hoc* test. **p <* 0.05, ***p <* 0.01, ****p <* 0.001. Differences with sex are indicated by a dagger symbol in **(B)**.

Supplementary Figure 6 Innate and T cell populations in the spleen. The spleen was removed and weighed. A portion, ~20mg, from the central region was removed, pressed through a 100µm strainer and cells collected and subjected to incubation with fluorescently labeled antibodies for flow cytometry. Flow cytometry was performed using the BD LSRFortessa Cell Analyzer and associated DIVA software. Data were analyzed using FlowJo software v10.10. Each dot indicates a sample from an individual mouse. These populations were identified and enumerated using the gating strategy shown in **Supplementary Figure 4**. **(A, C)** Spleen weight was indexed to body weight in male **(A)** and female **(C)** mice. Significance was calculated using ANOVA with Tukey’s *post-hoc* test (*p <* 0.05). Differences with sex are indicated by a dagger symbol in **(C)**. **(B, D)** Differences in absolute numbers of innate cells (first row) and T cells (second row) cells per femur in male **(B)** and female **(D)** mice between NoSx and SH or MI at different timepoints. Y-axis scales are matched to allow for easier comparisons of cell numbers between males and females. Significance was calculated using ANOVA with Tukey’s *post-hoc* test. **p <* 0.05, ***p <* 0.01, ****p <* 0.001. Differences with sex are indicated by a dagger symbol in **(D)**.
