# Supplementary figures and images for: Acute myocardial infarction induces sex-specific, time-dependent remodeling of the gut microbiome and intestinal immune compartment in retired breeder C57BL/6N mice
Source: Front Microbiomes. 2026 Jun 8;5:1818652. doi: 10.3389/frmbi.2026.1818652 (PMC13284139; doi:10.3389/frmbi.2026.1818652)

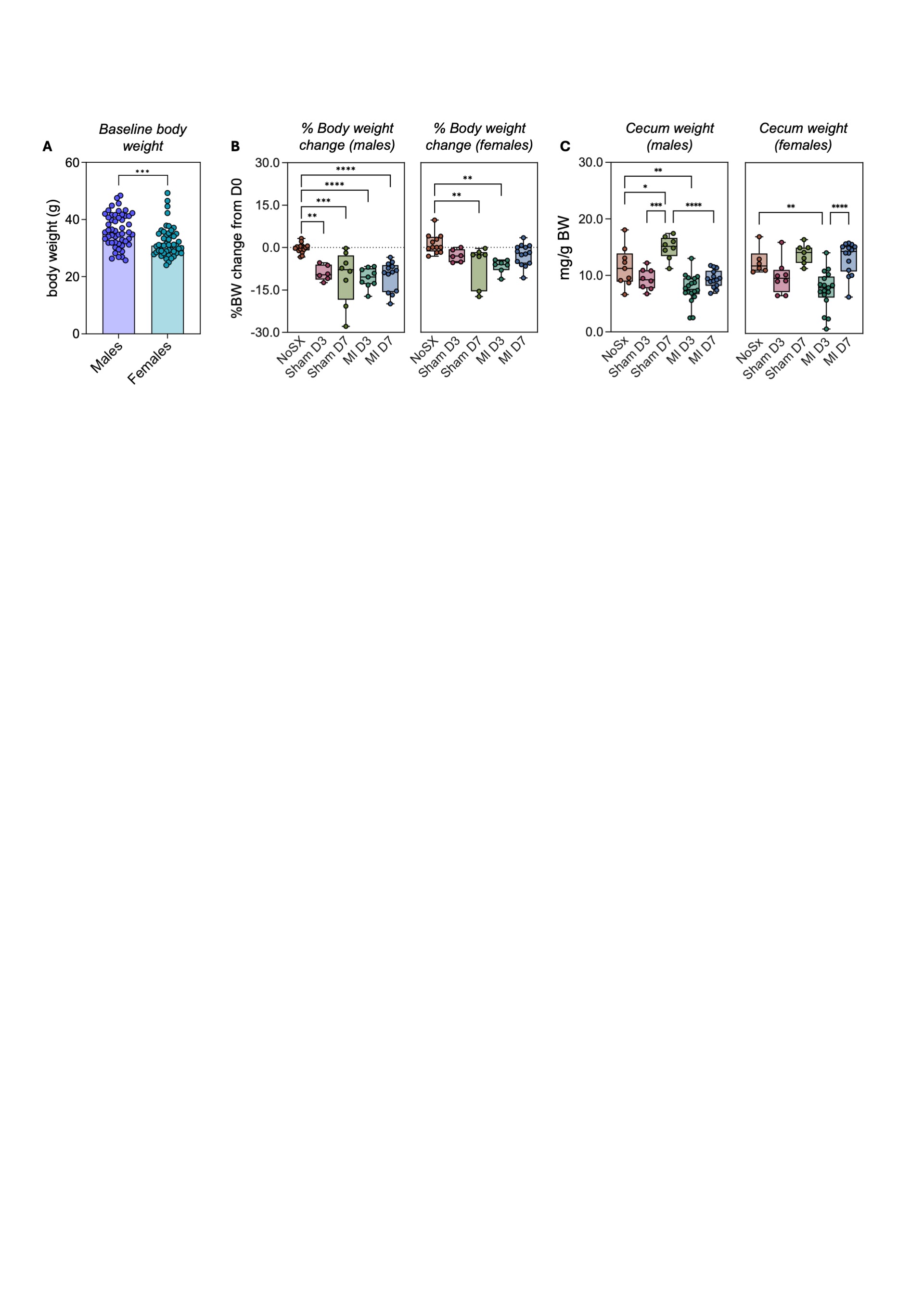

Supplement: Supplementary Figure 1 — Impact of surgery on total body and cecum weight. Mice were weighed before surgery and on day 3 (D3) or day 7 (D7) after surgery. At euthanasia, the cecum was dissected and contents weighed. Each dot indicates a sample from an individual mouse. (A). Actual body weights of male and female mice before surgery. Significance testing used an unpaired Student’s t-test. ***p < 0.001. (B) Percent body weight change (relative to NoSx controls) was in SH and MI mice at different timepoints after surgery. (C) Cecum weight indexed to body weight are shown. For B and C, significance was calculated using ANOVA with Tukey’s post-hoc test. *p < 0.05, **p < 0.01, ***p < 0.001, ****p < 0.0001. [file Image1.jpeg]

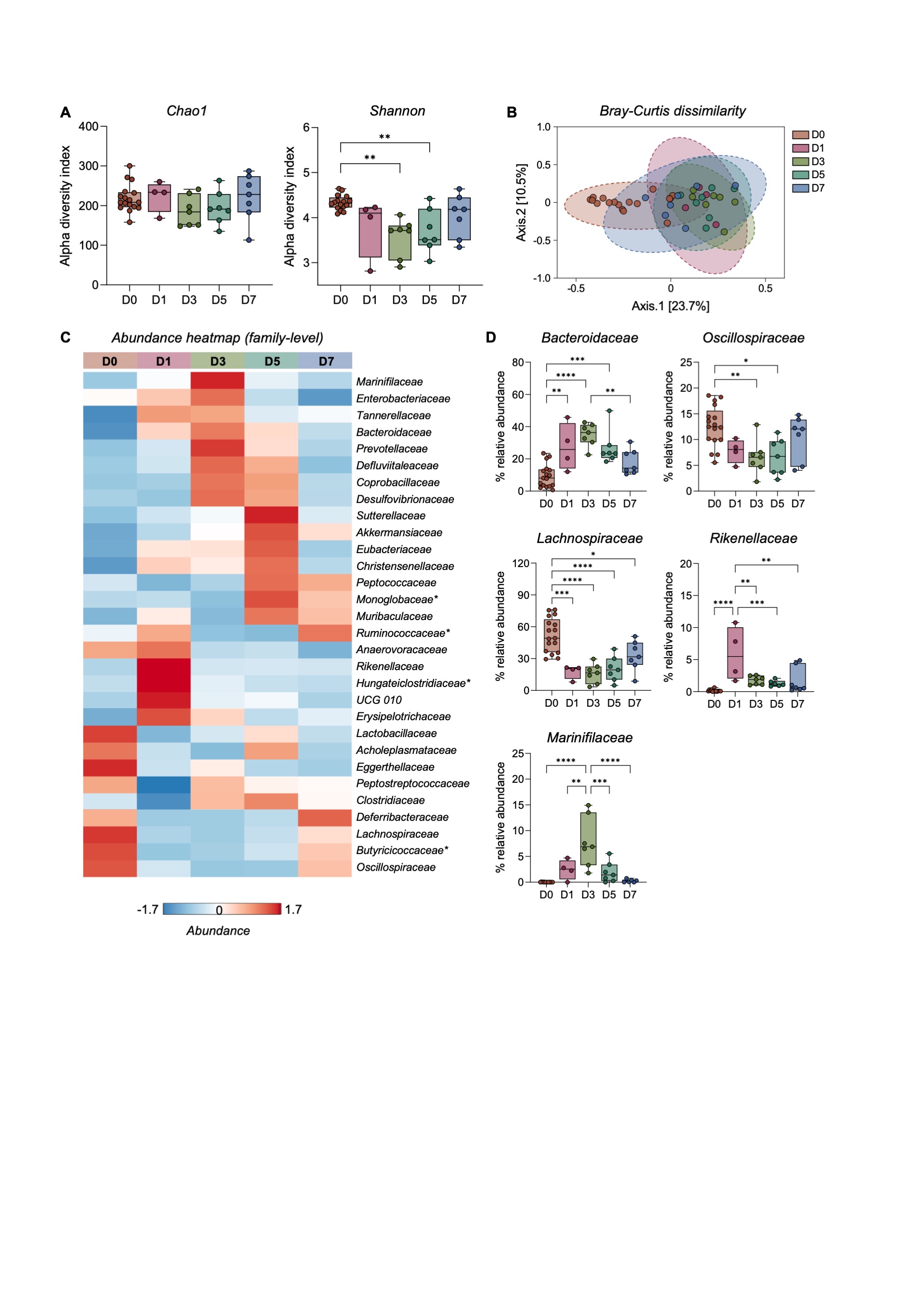

Supplement: Supplementary Figure 2 — Changes in gut microbiota at several different timepoints after MI in males. Fecal pellets were collected from male mice on the day of surgery (D0) and the indicated days after surgery. 16S rRNA sequencing of fecal DNA enabled microbiota profiling. Analyses were performed using MicrobiomeAnalyst v2.0. Each dot indicates a sample from an individual mouse. (A) Differences in alpha (within-sample) diversity after MI at different timepoints. Chao1 and Shannon indices of alpha diversity reflect taxonomic richness (Chao1 and Shannon) and evenness (Shannon) of microbiota. Significance was calculated using one-way ANOVA and Tukey’s post-hoc test. **p < 0.01. (B) Bray-Curtis Index with PERMANOVA was used to calculate beta (between-sample) diversity in microbiota samples collected at different timepoints after MI. Results are visualized by spatial clustering on PCoA plots. (C) A heatmap shows bacterial family relative abundance. Stars indicate members of the Oscillobacteraceae family. (D) Relative abundance of the most discriminative bacterial families in MI samples. Significance was calculated using ANOVA with Tukey’s post-hoc test. *p < 0.05, **p < 0.01, ***p < 0.001, ****p < 0.0001. [file Image2.jpeg]

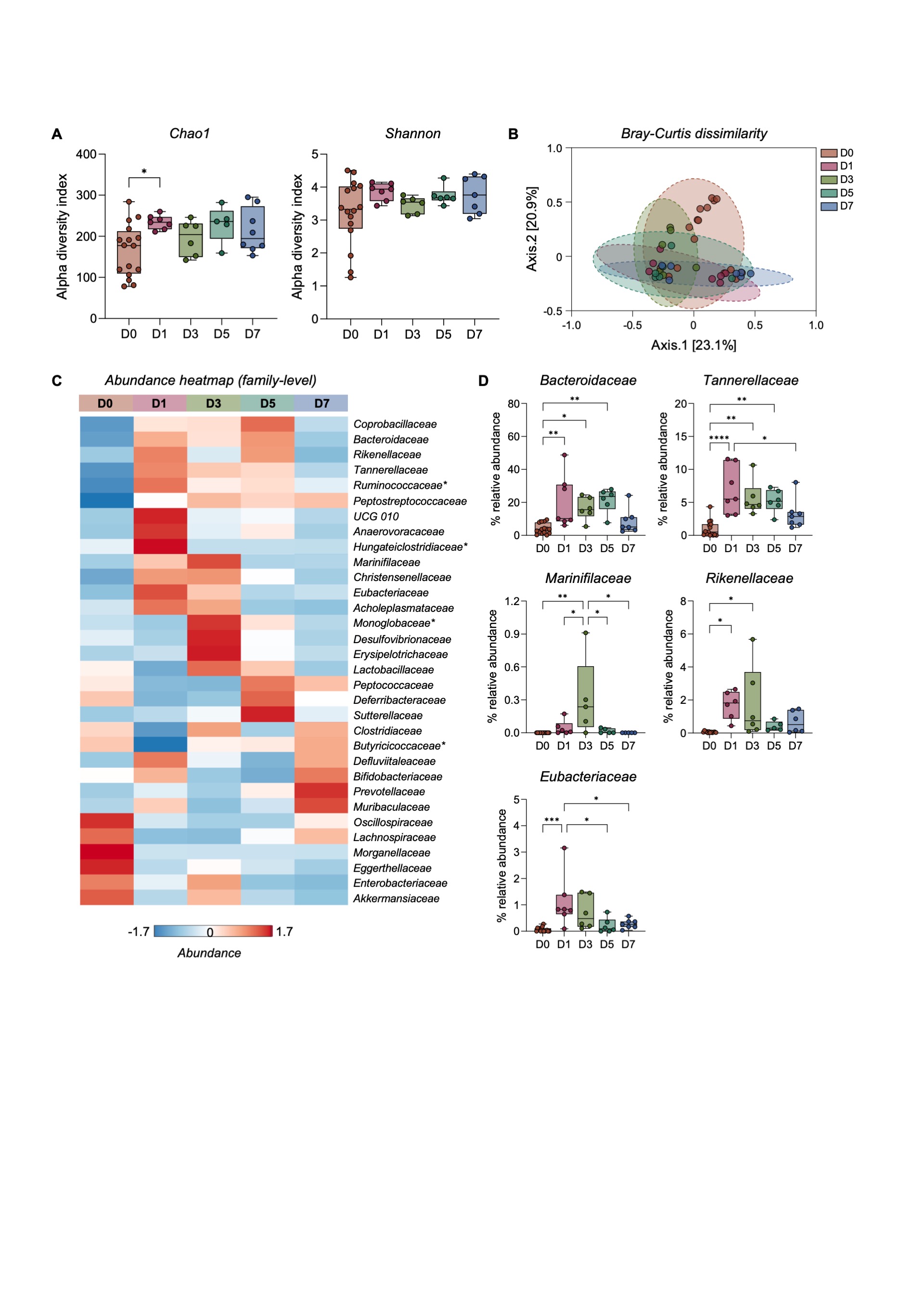

Supplement: Supplementary Figure 3 — Changes in gut microbiota at several different timepoints after MI in females. Fecal pellets were collected from female mice on the day of surgery (D0) and the indicated days after surgery. 16S rRNA sequencing of fecal DNA enabled microbiota profiling. Analyses were performed using MicrobiomeAnalyst v2.0. Each dot indicates a sample from an individual mouse. (A) Differences in alpha (within-sample) diversity after MI at different timepoints. Chao1 and Shannon indices of alpha diversity reflect taxonomic richness (Chao1 and Shannon) and evenness (Shannon) of microbiota. Significance was calculated using one-way ANOVA and Tukey’s post-hoc test (p < 0.05). (B) Bray-Curtis Index with PERMANOVA was used to calculate beta (between-sample) diversity in microbiota samples collected at different timepoints after MI. Results are visualized by spatial clustering on PCoA plots. (C) A heatmap shows bacterial family relative abundance. Stars indicate members of the Oscillobacteraceae family. (D) Relative abundance of the most discriminative bacterial families in MI samples. Significance was calculated using ANOVA with Tukey’s post-hoc test. *p < 0.05, **p < 0.01, ***p < 0.001, ****p < 0.0001. [file Image3.jpeg]

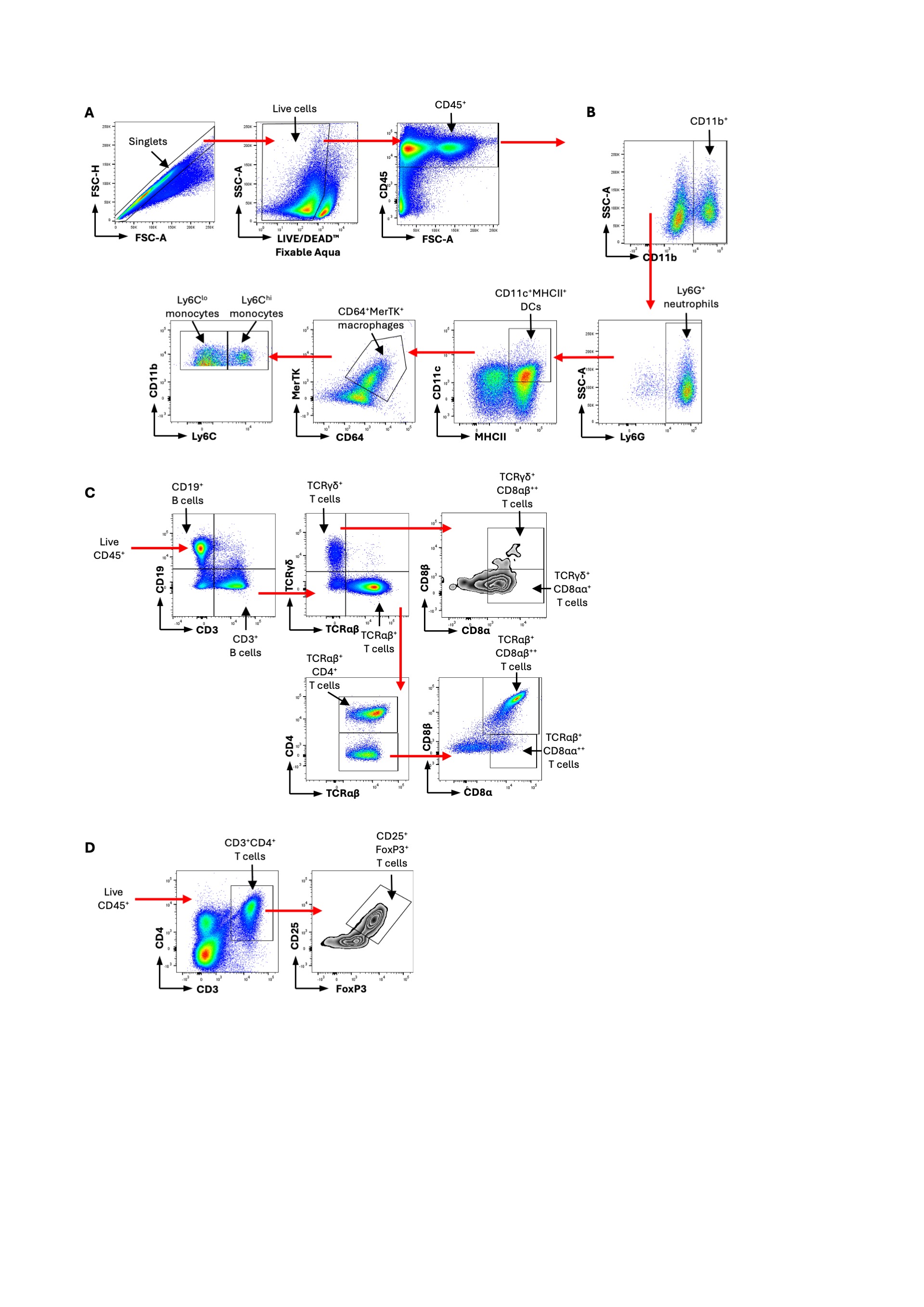

Supplement: Supplementary Figure 4 — Flow cytometry gating strategies. Flow cytometry was performed using the BD LSRFortessa Cell Analyzer and associated DIVA software. Data were analyzed using FlowJo software v10.10. The gating strategy to identify innate and T cell types in all tissues used. (A) Common gating strategy to isolate live, CD45+, single cell, immune cells in all panels. (B) Gating strategy to identify innate macrophage, monocyte, DC, and neutrophil immune cells. (C) Gating strategy to identify B cells, CD4+ and CD8+ T-cell subtypes. (D) Gating strategy to identify CD25+FoxP3+ Tregs. [file Image4.jpeg]

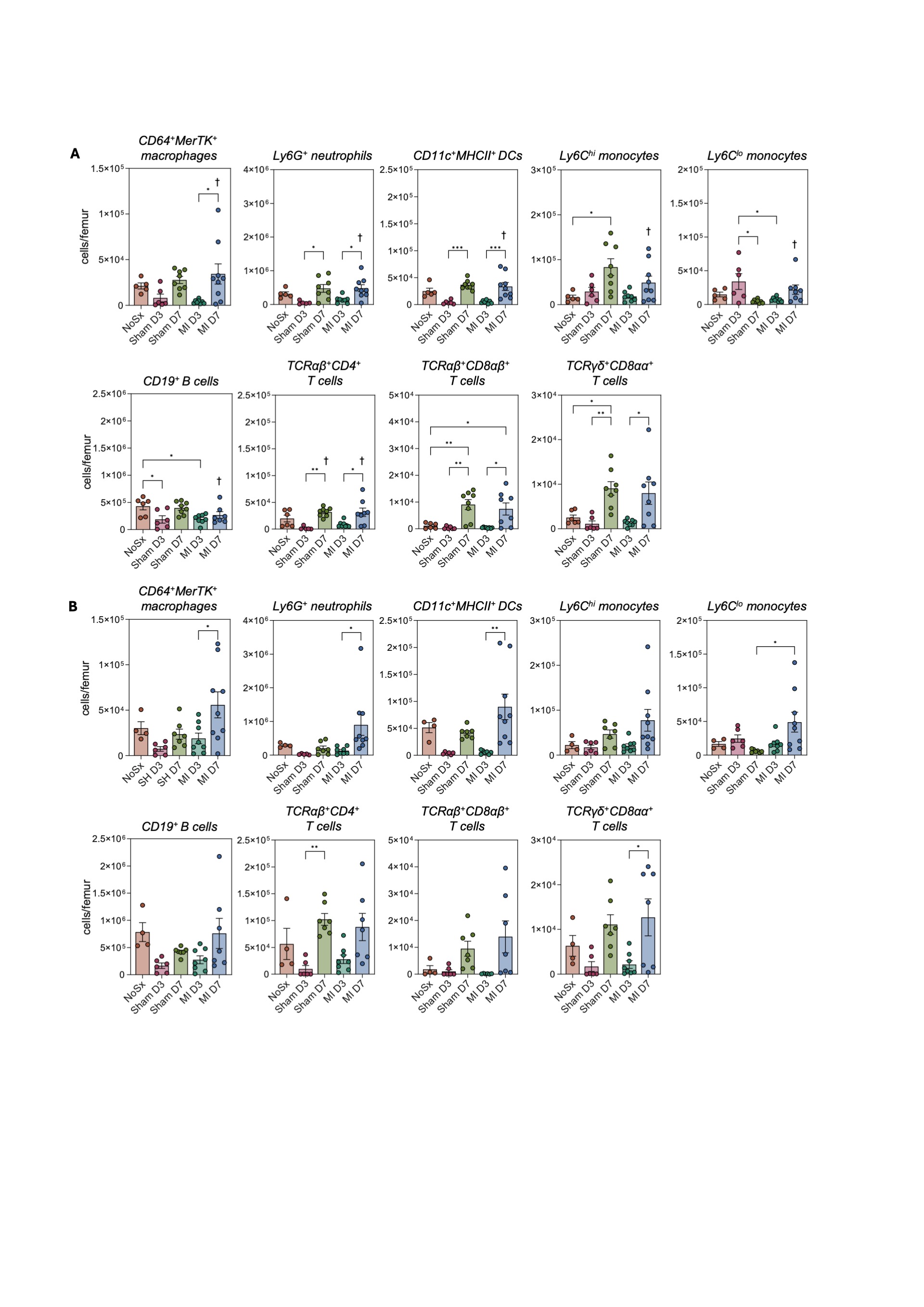

Supplement: Supplementary Figure 5 — Innate and T cell populations in the bone marrow. Bone marrow from the left femur was flushed and cells collected, filtered, and subjected to incubation with fluorescently labeled antibodies for flow cytometry. Flow cytometry was performed using the BD LSRFortessa Cell Analyzer and associated DIVA software. Data were analyzed using FlowJo software v10.10. Each dot indicates a sample from an individual mouse. These populations were identified and enumerated using the gating strategy shown in Supplementary Figure 4. (A, B) Differences in absolute numbers of innate cells (first row) and T cells (second row) cells per femur in male (A) and female (B) mice between NoSx and SH or MI at different timepoints. Y-axis scales are matched to allow for easier comparisons of cell numbers between males and females. Significance was calculated using ANOVA with Tukey’s post-hoc test. *p < 0.05, **p < 0.01, ***p < 0.001. Differences with sex are indicated by a dagger symbol in (B). [file Image5.jpeg]

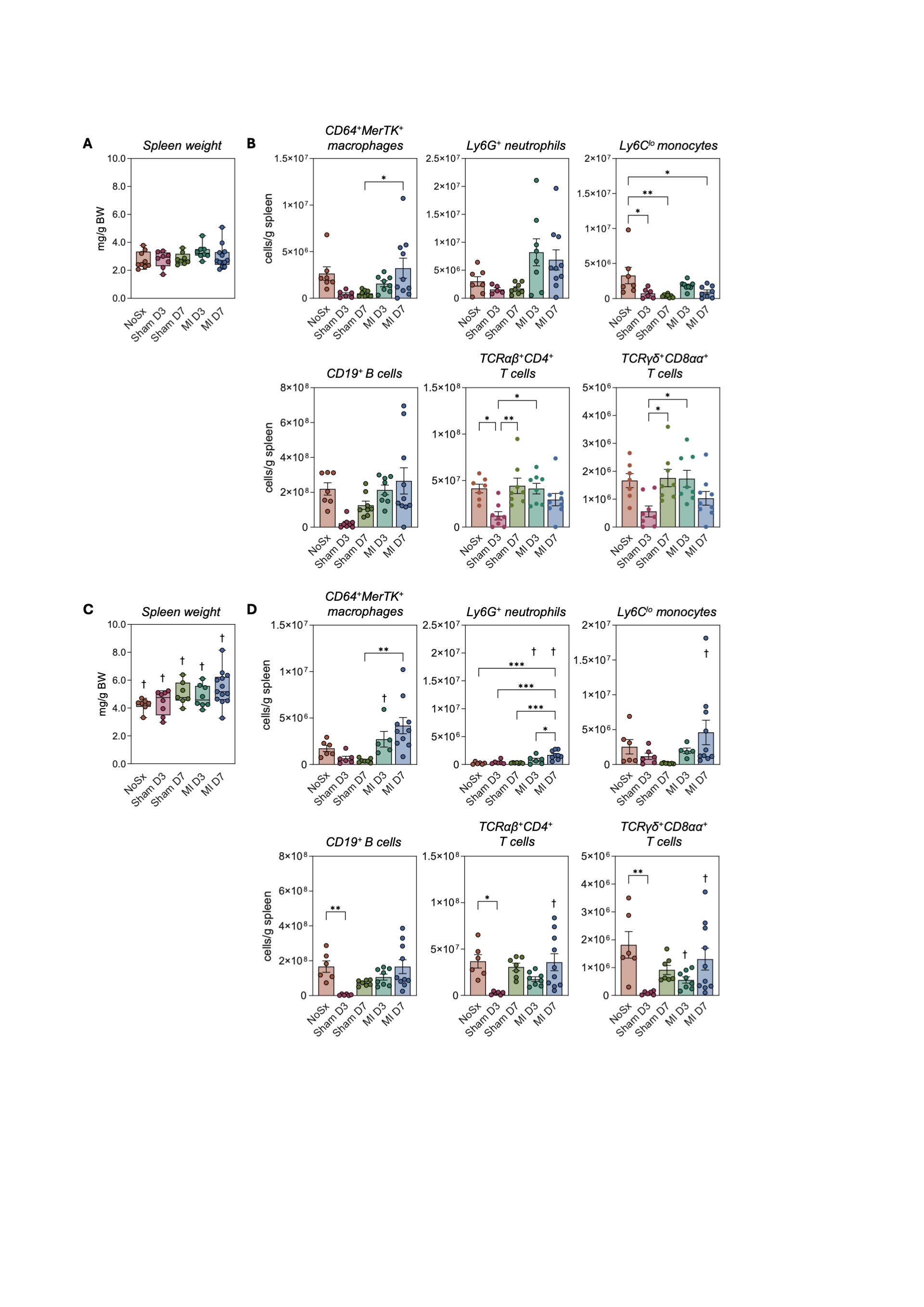

Supplement: Supplementary Figure 6 — Innate and T cell populations in the spleen. The spleen was removed and weighed. A portion, ~20mg, from the central region was removed, pressed through a 100µm strainer and cells collected and subjected to incubation with fluorescently labeled antibodies for flow cytometry. Flow cytometry was performed using the BD LSRFortessa Cell Analyzer and associated DIVA software. Data were analyzed using FlowJo software v10.10. Each dot indicates a sample from an individual mouse. These populations were identified and enumerated using the gating strategy shown in Supplementary Figure 4. (A, C) Spleen weight was indexed to body weight in male (A) and female (C) mice. Significance was calculated using ANOVA with Tukey’s post-hoc test (p < 0.05). Differences with sex are indicated by a dagger symbol in (C). (B, D) Differences in absolute numbers of innate cells (first row) and T cells (second row) cells per femur in male (B) and female (D) mice between NoSx and SH or MI at different timepoints. Y-axis scales are matched to allow for easier comparisons of cell numbers between males and females. Significance was calculated using ANOVA with Tukey’s post-hoc test. *p < 0.05, **p < 0.01, ***p < 0.001. Differences with sex are indicated by a dagger symbol in (D). [file Image6.jpeg]
